# Aerobic exercise facilitates p300 nuclear translocation via ADRB2-AMPKα signaling, leading to enhanced histone acetylation and mitigation of cognitive decline in APP/PS1 mice

**DOI:** 10.1186/s13195-026-01983-z

**Published:** 2026-02-10

**Authors:** Gao-shang Chai, Tian-long Gao, Shu-guang Bi, Yu-ming Mao, Liu Yang, Fang-zhou Wang, Jia Chen, Jia-jun Wu, Juan Gong, Shan Geng, Jia-qi Yuan, Ke-yu Zhang, Hai-yan Yi, Zi-chong Lan, Yun-juan Nie, Haitao Yu

**Affiliations:** 1 Department of Fundamental Medicine, Wuxi School of Medicine , Jiangnan University, Wuxi, Jiangsu 214122 P. R. China; 2 MOE Medical Basic Research Innovation Center for Gut Microbiota and Chronic Diseases, School of Medicine, Jiangnan University, Wuxi, Jiangsu China; 3 Department of electrophysiology, Wuhan Children’s Hospital (Wuhan Maternal and Children’s Healthcare Center), Tongji Medical College, Huazhong University of Science and Technology, Wuhan, Hubei China; 4 Department of Pathology, Affiliated Hospital of Jiangnan University, Wuxi, 214122 China

**Keywords:** Aerobic exercise, Histone acetylation, AMPKα, p300, Alzheimer’s disease, Synaptic plasticity

## Abstract

**Background:**

Physical activity (PA) is strongly associated with enhanced cognitive resilience and a lower risk of Alzheimer’s disease (AD) in the aging population. However, the molecular mechanisms linking exercise-induced neuroprotection to epigenetic remodeling remain poorly defined.

**Methods:**

A total of 1,511 participants from the National Health and Nutrition Examination Survey (NHANES) 2013–2014 cohort were included to assess the association between PA and cognitive performance. Mendelian randomization (MR) analysis was further employed to infer the causal relationship between PA and the risk of various dementias. Differential gene enrichment analysis was performed using the Gene Expression Omnibus (GEO) dataset (GSE110298) to compare transcriptomic profiles between sedentary and high PA groups in patients with AD. For mechanistic exploration, APP/PS1 transgenic mice underwent an 8-week treadmill-based aerobic exercise (AE) intervention (5 days/week, 40 min/day), followed by comprehensive assessments, including behavioral tests, pathological examinations, epigenetic and molecular biological analyses, and single-cell RNA sequencing.

**Results:**

Epidemiological analysis of the NHANES cohort revealed a nonlinear, dose-dependent relationship between PA and cognitive performance. MR supported a causal effect of genetically predicted higher PA on reduced AD risk. Transcriptomic profiling from GEO identified synaptic signaling and neurogenesis as key pathways modulated by exercise. In APP/PS1 mice, AE alleviated Aβ pathology and cognitive deficits, restored synaptic plasticity, and normalized synaptic protein expression. Mechanistically, AE activated ADRB2, triggering AMPKα phosphorylation and its interaction with the N-terminal (1–200 aa) region of p300. This interaction facilitated p300 nuclear translocation and subsequent enhanced histone H4K5 and H4K12 acetylation, promoting synaptic gene (e.g., GluN1) transcription. The AE-induced nuclear translocation of p300 and the improved synaptic plasticity in APP/PS1 mice were abolished by AMPKα inhibition with dorsomorphin (AMPK inhibitor, 10 mg/kg, intraperitoneal injection).

**Conclusion:**

These findings unveil a previously unrecognized ADRB2-p-AMPKα-p300 axis that AE utilizes to orchestrate chromatin remodeling, counteracting synaptic degeneration and cognitive decline in AD, providing actionable targets for exercise-mimetic therapies.

**Supplementary Information:**

The online version contains supplementary material available at 10.1186/s13195-026-01983-z.

## Introduction

Alzheimer’s disease (AD), the most widespread neurodegenerative disease and a primary contributor to dementia, represents an escalating international public health concern. Defined by a steady decline in cognitive performance, AD affects more than 55 million individuals worldwide, with forecasts suggesting the number will surpass 78 million by 2050 due to the global aging trend [1–5]. In neuropathological terms, AD is hallmarked by extracellular amyloid-β (Aβ) plaque, intracellular neurofibrillary tangles composed of hyperphosphorylated tau, synaptic deficits, and widespread neuronal loss, which collectively disrupt brain networks critical for memory and cognition [6–9]. Gene mutations (e.g., in *APP*, *PSEN1*, and *PSEN2*) cause fewer than 5% of Alzheimer’s cases. Most are sporadic AD, resulting from aging, genetic risks such as APOE ε4, environmental factors, and lifestyle [10]. Immunotherapy targeting Aβ and tau has achieved notable progress in AD. However, challenges such as the risk of cerebral hemorrhage and limited cognitive improvement remain [11]. Therefore, exploring interventions that ameliorate AD pathology and enhance cognitive function, particularly by promoting synaptic plasticity, represents a crucial strategic direction for AD prevention and treatment [12, 13].

Evidence suggests that epigenetic mechanisms, triggered by aging and environmental factors, link cognitive decline to impaired neuronal gene expression [14–17]. Histone acetylation, dynamically regulated by acetyltransferases (e.g., p300/CBP) and histone deacetylases (HDACs), maintains chromatin accessibility for synaptic plasticity-associated genes [18]. In AD, global histone hypoacetylation contributes to impaired neuroplasticity by downregulating key synaptic genes such as *bdnf*, glutamate receptor subunits *gria1* (glutamate receptor 1), *gria2*, *grin2a* (N-methyl D-aspartate receptor subtype 2 A), and *grin2b* [16, 19, 20]. This epigenetic “silencing” mechanism bridges aging to AD progression [21]. Epigenetic silencing is characterized by the accumulation of repressive histone marks, such as H3K27me3 and H3K9me3, and the loss of activating marks like H3K9ac, H3K14ac, H3K27ac, H4K5ac, and H4K12ac, leading to chromatin condensation and gene transcriptional repression [22]. This “silencing” is mediated by HDACs, which are increased in mouse models of neurodegeneration and in patients with AD [16]. Moreover, neuron-specific overexpression of HDAC2 impairs synaptic plasticity and compromises memory formation [16, 23]. On the other hand, several histone acetyltransferases (HATs), including p300/CBP, have been shown to be vital for neuronal processes such as synaptic plasticity and memory formation through reinstating histone acetylation homeostasis in the nucleus [24]. Dysregulation of HATs is strongly implicated in the pathogenesis of AD [25]. Notably, evidence suggests that the cytoplasmic retention of p300/CBP plays a role in the abnormal acetylation of tau protein, promoting its pathological aggregation. Simultaneously, a decrease in nuclear p300/CBP activity results in reduced histone acetylation in the brains of individuals with AD [25–28]. Therefore, targeting the subcellular mislocalization of p300/CBP, specifically by promoting its nuclear import and restoring histone acetylation, represents a promising therapeutic strategy for AD [29–33].

Aerobic exercise (AE) represents a potent lifestyle intervention for both the prevention and treatment of AD [34–36]. Epidemiological studies, including our own, demonstrate that regular AE reduces AD pathologies and attenuates cognitive decline [37–42]. Preclinical evidence indicates that AE ameliorates Aβ deposition, reduces tau hyperphosphorylation, and enhances mitochondrial function through augmented neurotrophic signaling (e.g., BDNF) and anti-inflammatory mechanisms [43]. However, the molecular pathways linking AE to epigenetic remodeling, particularly its effects on histone acetylation dynamics, remain poorly characterized. Critical questions persist: (1) Can AE rescue age- or AD-associated histone hypoacetylation to restore synaptic gene expression? (2) How do AE-induced signaling cascades mediate chromatin modifications? Addressing these knowledge gaps carries profound therapeutic implications for the cognitive preservation of individuals with neurodegenerative diseases.

The β2-adrenergic receptor (ADRB2) is a type of G protein-coupled receptor (GPCR) that primarily binds to epinephrine and norepinephrine, playing a key role in regulating various physiological functions and metabolic processes in the body. In the brain, ADRB2 is expressed on the plasma membranes of neurons and glial cells, where it participates in regulating neural function and cerebral metabolic processes [44, 45]. ADRB2 mediates AE-induced neuroadaptations and is essential for AE-driven hippocampal neurogenesis, suggesting a key role in bridging metabolic stimuli to epigenetic regulation [34, 43, 46–48]. ADRB2 activation during exercise induces cyclic AMP (cAMP) signaling, which converges with AMP-activated protein kinase (AMPK) pathways, a master metabolic sensor activated by energy fluctuations [49]. Notably, the dysfunction of AMPK signaling is implicated in AD development by regulating energy metabolism, autophagy, mitochondrial function, and other mechanisms [50, 51]. Moreover, activation of AMPK could mitigate Alzheimer-related dendritic abnormalities and memory deficits [21, 52, 53]. However, the precise role of AMPK in mediating neuronal chromatin remodeling, particularly in the context of exercise-induced histone acetylation dynamics, remains to be fully elucidated.

Although epidemiological studies link PA to reduced AD risk, the underlying neuroprotective mechanisms remain unclear. We focus on epigenetic dysregulation, specifically the impaired histone acetylation caused by the aberrant localization of the acetyltransferase p300. We hypothesize that AE activates ADRB2-AMPKα signaling, promoting the nuclear translocation of p300, which restores its role in histone acetylation and corrects synaptic gene expression. We further explore the direct interplay between AMPKα and p300 that facilitates this process. Our work aims to delineate a novel ADRB2-AMPKα-p300 axis, providing mechanistic insights into exercise-induced neuroprotection and paving the way for innovative therapeutic strategies.

## Materials and methods

### Observational study design

This study utilized data from the 2013–2014 cycle of the NHANES, and included 1,511 individuals who underwent the Digit Symbol Substitution Test (DSST) and for whom comprehensive records of PA and relevant covariates were available. The DSST served as a tool to evaluate cognitive function, primarily targeting domains such as processing speed and executive functioning. Information on PA was self-reported through questionnaires that encompassed five activity domains, including vigorous/moderate-intensity work, transportation (e.g., walking or biking), and recreational activities. Weekly energy expenditure for each activity type was estimated in MET-minutes using the equation: frequency (days/week) × duration (minutes/day) × assigned MET coefficient, where MET values of 8.0 and 4.0 were applied to vigorous-and moderate-intensity activities, respectively. Aggregating these values yielded the overall PA level per participant. Adjustments were made for a range of covariates, such as demographic factors (age, sex, race/ethnicity), socioeconomic status (education), and health-related behaviors (Body Mass Index, smoking, alcohol use). Sampling weights reflecting NHANES’s complex survey design were incorporated into all analyses. In line with previous studies [3, 54], both multivariate linear models and restricted cubic spline (RCS) approaches, with three pre-specified knots, were employed to investigate the nonlinear association between total MET and DSST scores. All statistical procedures were implemented in R (version 4.4.0), considering a two-tailed *P* value of < 0.05 as statistically significant.

### Mendelian randomization (MR)

We utilized a two-sample MR strategy to investigate the potential causal link between strenuous PA and five distinct dementia subtypes. The genetic summary data for the exposure originated from a genome-wide association study (GWAS) on strenuous exercise and sports (GWAS ID: ebi-a-GCST006100), published in 2018. Instrumental variables were stringently filtered based on genome-wide significance (*P* < 5 × 10⁻⁸), low linkage disequilibrium (r² < 0.001), and a clumping window of 10,000 kilobases. GWAS summary data corresponding to five dementia types were employed as outcomes: AD (21,982 cases / 41,944 controls), vascular dementia (881 / 211,508), frontotemporal dementia (515 / 2,509), dementia with Lewy bodies (2,591 / 4,027), and overall dementia (5,933 / 212,859). The main analysis adopted the inverse-variance weighted (IVW) estimator within a random-effects framework to infer causality. Additional sensitivity evaluations included MR-Egger regression, weighted median estimator, and MR-PRESSO to verify the consistency of the results. Heterogeneity was assessed using Cochran’s Q test, and leave-one-out approaches were applied to identify the influence of single nucleotide polymorphisms (SNPs). Reverse MR was conducted to rule out reverse causation. All statistical analyses were carried out in R (version 4.4.0) using the TwoSampleMR toolkit and associated packages to ensure replicability and robustness.

### Transcriptomic analysis

The transcriptomic dataset GSE110298 was retrieved from the GEO repository. Expression profiles and corresponding clinical data were preprocessed in R (v4.3.2), and samples were categorized according to PA levels assessed by wrist-worn accelerometers. DEGs between the high- and low-activity cohorts were determined using the limma package with significance cutoffs of *P* < 0.05 and |log₂fold change| > 0.58. Subsequently, functional enrichment analysis based on Gene Ontology (GO) terms was performed using the clusterProfiler package. GSEA was performed using curated gene sets obtained from the Molecular Signatures Database (MSigDB, https://www.gsea-msigdb.org/gsea/msigdb). To further explore molecular interactions, protein–protein interaction (PPI) networks of the identified DEGs were generated from the STRING database (https://cn.string-db.org/) and visualized with Cytoscape (v3.10.1). In addition, Bayesian network modeling implemented via the CBNplot package (https://github.com/noriakis/CBNplot) was used to infer potential causal relationships among enriched genes and pathways, offering deeper insight into the molecular mechanisms through which PA may influence AD.

### Mice and aerobic exercise (AE)

Each experimental group included mice allocated for specific downstream analyses: two hemispheres from two mice were used for scRNA-seq, two hemispheres from two mice for TEM, six hemispheres from six mice for Western blotting, RT–PCR, and ChIP assays, and three hemispheres from three mice for Golgi staining. All animal procedures received approval from the Animal Ethics Committee of Jiangnan University (JN.No.20240330t0320731). The eight-month-old male transgenic B6C3-Tg (APPswe, PSEN1dE9) mice (with pathological and cognitive impairment starting from 6 to 8 months) [46], sourced from the Nanjing University Model Animal Center, were employed as AD models. Age- and sex-matched WT B6C3 mice served as control subjects. All mice were housed under specific-pathogen-free (SPF) conditions at the Experimental Animal Center of Jiangnan University. Beginning at 8 months of age, animals in the AE group undertook treadmill running (TECHMAN, Chengdu, China) for 5 days per week, 40 min/day, over an 8-week period. The training regimen followed a progressive protocol with increasing speeds: 6 m/minute for 5 min, 9 m/minute for 5 min, 12 m/minute for 20 min, 15 m/minute for 5 min, and 12 m/minute for another 5 min, all on a 0 incline. A motivating electrical stimulus (1 mA) was delivered to keep mice running. Meanwhile, sedentary mice were placed in the same exercise environment without treadmill activity, ensuring consistent exposure to experimental conditions. Observational assessments were performed during sessions to ensure adherence to the exercise routine. In the AMPKα inhibition experiment, APP/PS1 mice received intraperitoneal injections of the AMPK antagonist Compound C (also known as dorsomorphin; TSBiochem, T1977), a selective and ATP-competitive AMPK inhibitor, at a dose of 10 mg/kg body weight, 1 h prior to the initiation of AE. Mice in the WT and APP/PS1 + AE groups received equivalent volumes of vehicle solution (5% DMSO in 0.9% NaCl) at the same time point.

### Behavioral testing

Behavioral experiments were conducted between 1:00 p.m. and 5:00 p.m. after the completion of the aerobic training period. In the MWM test, the water was maintained at 22–23 °C and made opaque with the addition of milk powder. The water level was kept 2 cm above the surface of a submerged escape platform. The pool was divided into four quadrants, each marked with distinct visual cues. During the acquisition phase (days 1–6), mice underwent four trials per day, with each trial starting from a different, randomly assigned quadrant. Mice were released from the pool wall and given 60 s to locate the hidden platform; successful mice remained on the platform for 30 s. On Day 7, a probe trial was conducted in which the platform was removed, and mice were allowed to swim freely for 60 s, starting from the quadrant opposite the platform’s original location. Performance measures included swim path, escape latency, number of platform crossings, and time spent in the target quadrant, all recorded via an automated video tracking system (FCT-100, TECHMAN). The contextual freezing test was conducted on the first two days after the MWM test to assess contextual memory. Mice were placed in a chamber with an electrifiable grid floor inside a soundproof box. In the conditioning phase, each mouse acclimated for 3 min before receiving 3 foot shocks (1 mA, 2 s) at 58-second intervals. The chamber was cleaned with 75% ethanol between trials. In the testing phase, mice were returned to the chamber for 3 min without shocks after 2 h. Freezing time was recorded by an overhead camera and analyzed using FCT-100 software (Taimeng, China). Immediately after the completion of the behavioral tests, mice were either sacrificed for brain tissue collection or subjected to transcardial perfusion with 0.9% sodium chloride followed by 4% paraformaldehyde for fixation, according to subsequent experimental requirements.

### Tissue extraction

Upon completion of behavioral testing, animals were anesthetized via intraperitoneal injection of 20% urethane (10 mL/kg). Prior to the procedure, all surgical instruments, perfusion equipment, and padding materials were sterilized and prepared. Anesthesia depth was confirmed by the absence of the pedal withdrawal reflex. Subjects were then immobilized on a foam platform, followed by rapid thoracotomy. Cardiac perfusion was performed through the left ventricular apex using a 20 mL pre-warmed (37 °C) 0.9% sodium chloride solution over 3 min. Immediately post-perfusion, brains were extracted via craniotomy. The left hippocampus was dissected on a chilled platform and flash-frozen in liquid nitrogen for storage at − 80 °C pending molecular analysis. The contralateral hemisphere was immersion-fixed in a 4% paraformaldehyde (PFA) solution for 24 h at 4 °C for histopathological examination.

### Cell culture and treatment

N2a and 293T cell lines were maintained in DMEM (SH30243.01, Cytiva, Marlborough, MA, USA) containing 10% FBS (BS1614-109, Bioexplorer, USA) and 1× penicillin–streptomycin solution (BL505A, Biosharp, Anhui, China) under standard culture conditions (37 °C, 5% CO₂). Cells were seeded onto well plates and grown to approximately 70% confluence before subsequent treatments. For ADRB2 activation experiments, cells were treated with the ADRB2 agonist Teb (MedChemExpress, Monmouth Junction, NJ, USA, HY-B0802A) at a concentration of 10 µM for 1 h. Where indicated, AMPK signaling was inhibited by co-treatment with dorsomorphin (TSBiochem, T1977) at 10 µM.

### Western blotting

Total protein was isolated from hippocampal tissue using RIPA buffer supplemented with a protease inhibitor cocktail. Protein concentration was determined using a BCA assay kit (E112, Novozan, China). Samples (20 µg/lane) were resolved by 10% SDS-PAGE and electrophoretically transferred to NC membranes (0.45 μm; Millipore, Burlington, MA, USA). After blocking with 5% bovine serum albumin (BSA) in Tris-buffered saline with 0.1% Tween-80 (TBST) for 1 h at 25 °C, membranes were incubated with primary antibodies (Table 1 for specifications) overnight at 4 °C. Following three 10-minute TBST washes, membranes were probed with HRP-conjugated secondary antibodies (1:10,000 dilution; Cell Signaling Technology) for 1 h at room temperature. Chemiluminescent signals were developed using ECL substrate (Thermo Fisher Scientific, Waltham, MA, USA) and quantified by densitometry (ImageJ v1.53). β-actin served as the loading control for protein normalization.

**Table 1:** Antibodies used in WB and IF

Antibody	Specificity	Type	Dilution for WB	Source	Catalog number
β-actin	β-actin	Poly-	1:1000	SAB	21,335
AMPKa1	AMPK alpha 1	Mono-	1:1000	SAB	48,827
p-AMPKα	Phospho-AMPKα (Thr172)	Mono-	1:1000	CST	2535
p300	p300	Mono-	1:1000 1:100 for IP	CST	70,088
P-p300	p300 (phospho-Ser89)	Poly-	1:1000	SAB	13,650
ADRB2	Beta 2 adrenergic receptor	Mono-	1:1000	Abcam	182,136
GluN2A	NMDAR2A	Poly-	1:1000	SAB	53,091
GluN2B	NMDAR2B	Poly-	1:1000	SAB	54,739
GluN1	NMDAR1	Poly-	1:1000	SAB	49,488
SYN1	Synapsin-1	Poly-	1:1000	SAB	41,470
PSD95	PSD95	Poly-	1:1000	SAB	45,221
H3K14	Histone H3 acetylated at lysine 14	Poly-	1:1000	Abcam	ab232952
H3K18	Histone H3 acetylated at lysine 18	Mono-	1:10000	Abcam	ab40888
H3	Histone H3	Poly-	1:1000	SAB	41,017
H4K12	Histone H4 acetylated at lysine 12	Poly-	1:5000	Abcam	ab46983
H4K5	Histone H4 acetylated at lysine 5	Mono-	1:10000	Abcam	ab51997
H4	Histone H4	Mono-	1:1000	Abcam	ab177840
FLAG	Flag-Tag	Mono-	1:1000	SAB	T519
p300	p300	Mono-	1:100 for IF	Immunoway	YM8860

### Co-IP

Hippocampal tissues (20 mg) were homogenized in ice-cold IP lysis buffer (Beyotime Biotechnology, Shanghai, China, P0013B) using a cryogenic homogenizer, followed by centrifugation at 14,000 × *g* for 5 min at 4 °C. The supernatant (100 µL) was incubated with Protein A/G magnetic beads (20 µL; Beyotime Biotechnology, P2055) that had been pre-incubated with 2 µg of primary antibody for 24 h at 4 °C with rotation. Beads were washed three times with PBS (pH 7.4) and subsequently incubated with tissue lysate overnight at 4 °C. Prior to incubation, the antibody-conjugated magnetic beads were subjected to three sequential washes with phosphate-buffered saline (PBS, pH 7.4) for 30 s per wash under centrifugation at 4,000 rpm. Co-precipitated proteins were detected by Western blot using anti-rabbit IgG secondary antibodies (Vazyme Biotech, Nanjing, China, 7E710H3) to minimize IgG interference.

### Cytosolic/nuclear fractionation and Immunoblot analysis

Nuclear and cytoplasmic proteins were isolated from N2a cells (Probiotic, CL0168, China) or hippocampal tissues using a subcellular protein extraction kit (Beyotime Biotechnology, P0028). Briefly, cells/tissues were first lysed in cytoplasmic extraction buffer containing protease inhibitors. Then, nuclear pellets were obtained by centrifugation at 14,000 × *g* for 10 min. Subsequently, nuclear proteins were extracted with nuclear extraction buffer. Finally, protein fractions (20 µg per lane) were resolved by 10% SDS-PAGE and transferred to NC membranes.

### Reverse transcription and real-time qPCR

Total RNA was extracted from hippocampal and cortical tissues using TRIzol reagent. Briefly, tissues were homogenized with a cryogenic homogenizer (Jingxin, Shanghai, China), and RNA was isolated through phase separation with chloroform, isopropanol precipitation, and 75% ethanol washing steps. The RNA pellet was air-dried and resuspended in 50 µL RNase-free water. Reverse transcription was performed using a reverse transcription kit (Vazyme Biotech, R312–01) on a Mastercycler PCR instrument (Eppendorf, Hamburg, Germany). Real-time qPCR was conducted on a LightCycler 480 II system (Roche Life Science, Basel, Switzerland) using SYBR Green reagents (YEASEN, Shanghai, China) following the manufacturer’s protocols. Primer sequences employed for qPCR are detailed in Table 2.

**Table 2:** Primers employed in RT-qPCR

Gene	Forward primer (5’ → 3’)	Reverse primer (5’ → 3’)
PSD95	ACCCTAGAAGCCCCAGGATA	AAGCCCAGACCTGAGTTACC
GluN1	CACAGAAGTGCGATCTGGTGAC	GGCATTGCTGCGGGAGT
GluN2A	TTGGGAGCGGGTACATCTTT	CTCCTGCCATGTTGTCGATG
GluN2B	GGAGAGGGTGGGAAAATGGA	ACAATGACAAACGGTGCCTC
Chip-GluN1	TGTGCAGACATGGAACCTC	AGAATCCTGGACCCACACG

### Golgi staining

Mice were deeply anesthetized by intraperitoneal injection of 20% ouabain, followed by rapid brain extraction and surface blood vessel rinsing with distilled water. Whole brains were immersed in freshly prepared A + B impregnation solution (Rapid Golgi Stain kit, FD Neuro Technologies, Columbia, MD, USA, PK40) and stored in light-proof containers for 14 days. After incubation in Solution C at 4 °C for 48 h, tissues were sectioned coronally at 100 μm using a cryostat. Sections were stained in D + E solution for 10 min at room temperature, dehydrated through a graded ethanol series, and imaged using a Zeiss Axio Imager Z2 microscope. Dendritic spine density and Sholl analysis were performed using ImageJ/Fiji software, with ≥ 3 secondary dendrites analyzed per neuron.

### scRNA-seq, quality control, cell clustering, and dataset integration

We extracted samples from the hippocampal tissue of WT, APP/PS1, and APP/PS1 + AE mice and quickly prepared single-cell suspensions. Processed samples were delivered to NovelBio Laboratory (www.novelbio.com) for 10x Genomics single-cell sequencing. Raw sequencing files were subjected to preliminary quality filtering, followed by dimensional reduction analysis using PCA and UMAP for data visualization and quality assessment. After preliminary filtering, quality control was performed using PCA and UMAP, and further filtering was conducted based on the number of genes, total counts, and mitochondrial gene percentage. The filtering criteria were set to include gene counts between 200 and 4,000, with a mitochondrial gene expression ratio of less than 10%. The data were then normalized using the LogNormalize method, and PCA dimensionality reduction was followed by UMAP visualization. To correct for batch effects, the Harmony method was used to ensure data consistency. Cell clustering was performed using FindNeighbors and FindClusters, followed by cell type annotation using the SingleR reference database. Differential gene analysis was conducted using the FindMarkers function, with the Wilcoxon rank-sum test and FDR correction, and enrichment analysis was performed using the limma package.

### Chromatin Immunoprecipitation (ChIP)

The ChIP procedure was carried out using a commercial kit (P2078, Beyotime Biotechnologies) following the manufacturer’s instructions. Mouse hippocampal tissues were fixed with formaldehyde at room temperature for 15 min to crosslink DNA and proteins, and the reaction was stopped with glycine for 10 min. After two washes with ice-cold PBS, the tissues were sonicated on ice using an ultrasonic processor (IID, Scientz, China). The clarified supernatant was diluted in ChIP buffer and pre-cleared with Protein A/G agarose beads containing salmon sperm DNA to minimize nonspecific binding. Following centrifugation, samples were immunoprecipitated with target-specific antibodies (optimally diluted) overnight at 4 °C with rotation. Immune complexes were subsequently captured by fresh Protein A/G agarose beads and washed through sequential salt gradients, culminating in a final TE buffer wash (50 mM Tris-HCl, 10 mM EDTA). Precipitated DNA was purified (Beyotime Biotechnologies, D0033) and analyzed by RT-qPCR using the LightCycler 480 system (Roche Life Science).

### Immunofluorescence staining

N2a cells (CL0168, Probiotic China) were cultured on confocal dishes until.

70–80% confluency, then treated with Teb (1 h) and washed with PBS. After fixation with 4% PFA (4 °C for 20 min → RT for 5 min) and PBS washes, cells were permeabilized with 0.5% Triton X-100 (RT, 20 min), blocked with 5% goat serum (1 h), and incubated with primary antibodies (4 °C overnight). Following PBS washes, Alexa Fluor-conjugated secondary antibodies (1:200) were applied (37 °C, 1 h) and then subjected to confocal imaging (Zeiss Axio Imager Z2).

### TEM

Following the completion of the MWM test, mice were euthanized and transcranially perfused with 3% paraformaldehyde and 2% glutaraldehyde in PBS. Hippocampal tissues were rapidly isolated and fixed in 2.5% glutaraldehyde for at least 4 h. Samples were submitted to Shiyanjia Laboratory (www.shiyanjia.com) for further processing. Tissues were dehydrated through a graded ethanol series (30%, 50%, 70%, 80%, 90%, 95%, and 100%), with each step lasting approximately 30 min, followed by immersion in absolute acetone for 30 min. They were then incubated in a 1:3 mixture of absolute acetone and Spurr resin for 3 h and subsequently infiltrated overnight in pure Spurr resin. Polymerization was carried out at 70 °C for over 9 h. Ultrathin sections were stained for 5–10 min and examined under a Hitachi H-7650 TEM (Hitachi, Japan).

### Antibodies and plasmids

The antibodies employed in this study are shown in Table 1.

The plasmids used in this study are as follows: Flag-EP300, EP300 1–90 pcDNA3.1-3xFlag-C, EP300 91–200 pcDNA3.1-3xFlag-C, EP300 201–300 pcDNA3.1-3xFlag-C, EP300 301–2000 pcDNA3.1-3xFlag-C, EP300 2001–2414 pcDNA3.1-3xFlag-C, EP300 del 1–90 pcDNA3.1-3xFlag-C, EP300 del 91–200 pcDNA3.1-3xFlag-C, and EP300 del 1-200 pcDNA3.1-3xFlag-C, which were obtained from UNIBIO Biological Technology Co. (Changsha, China).

### Statistical analysis

Quantitative data were expressed as means ± SEMs. MWM training data and Sholl analysis were analyzed using two-way repeated-measures ANOVA followed by Bonferroni’s post hoc test. Additionally, probe trial results, fear conditioning performance, and molecular biology data (Western blot, RT-PCR, immunofluorescence) were evaluated using one-way ANOVA followed by Bonferroni’s post hoc test. Normality (Shapiro–Wilk test) and homogeneity of variance (Levene’s test) were assessed prior to applying parametric tests; when assumptions were violated, Welch’s ANOVA or the Kruskal–Wallis test was used as appropriate. All statistical computations were conducted using GraphPad Prism 9 (GraphPad Software, San Diego, CA, USA), with *P* < 0.05 considered statistically significant.

## Results

### Physical activity (PA) reduces AD risk and enhances cognitive function by promoting synaptic plasticity in individuals with AD

**Fig. 1:**
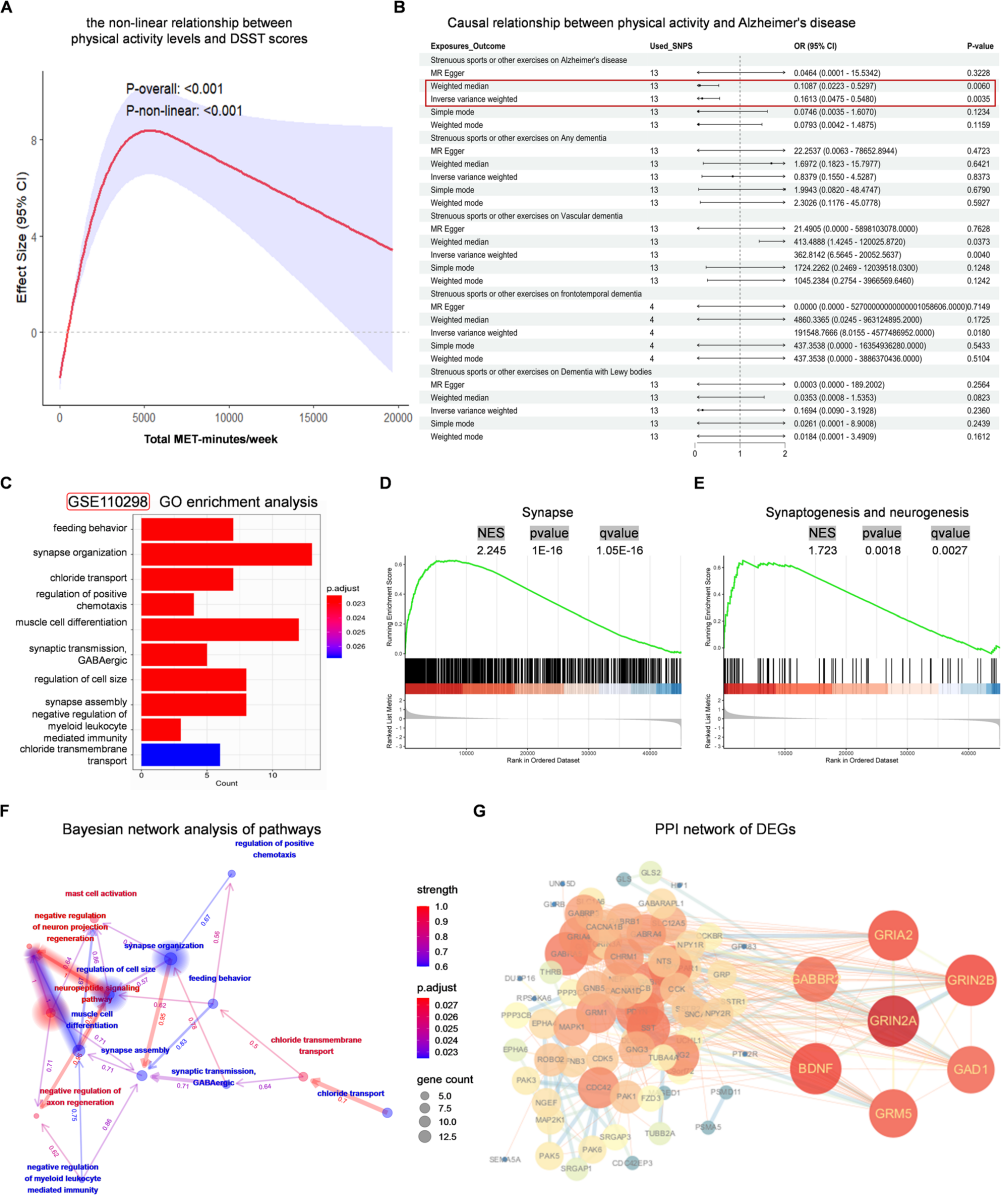
Physical activity reduces AD risk and enhances cognitive function by promoting synaptic plasticity in individuals with AD. **A** RCS curve of the dose-response relationship between physical activity and cognitive function: Cross-sectional analysis from the NHANES database showing the non-linear relationship between physical activity levels and DSST scores using restricted cubic splines (RCS). **B** Causal relationship between physical activity and Alzheimer’s disease: Mendelian randomization analysis assessing the relationship between physical activity and the risk of various types of dementia. **C** Differential gene enrichment analysis in Alzheimer’s disease patients: Differential gene enrichment analysis comparing sedentary and high physical activity individuals in AD patients from the GEO database (GSE110298). **D** and **E** Gene set enrichment analysis (GSEA) of differentially expressed genes (DEGs): Enriched biological pathways related to synaptic function, neurogenesis, and neurodegenerative changes in AD. **F** Bayesian network analysis of pathways from (C) GO enrichment analysis: Identification of key molecular pathways associated with AD progression. **G** Protein-protein interaction (PPI) network of DEGs: PPI network of DEGs revealing key proteins involved in synaptic plasticity and neurogenesis

To investigate the relationship between PA, cognitive performance, and the likelihood of developing AD, we performed comprehensive analyses integrating multiple large-scale datasets. Initially, we analyzed data from the 2013–2014 cycle of the National Health and Nutrition Examination Survey (NHANES). A restricted cubic spline (RCS) model demonstrated a significant non-linear association between levels of PA, measured in metabolic equivalent (MET) minutes per week, and their estimated effects on Digit Symbol Substitution Test (DSST) scores, indicating that the cognitive benefits of PA are not uniform across all activity levels (*P*-overall < 0.001; *P*-non-linear < 0.001; Fig. 1A). The analysis suggested that cognitive performance improves with increased PA, reaching an optimal threshold beyond which additional activity yields diminishing returns. To further explore potential causal links between PA and dementia risk, we employed Mendelian randomization (MR) with summary statistics derived from the OpenGWAS database. The inverse variance weighted (IVW) method demonstrated that genetically predicted higher PA levels were significantly associated with a reduced risk of AD (odds ratio [OR] = 0.1613; 95% confidence interval [CI]: 0.0475–0.548; Fig. 1B). However, no significant causal associations were identified between PA and the risks of vascular dementia, frontotemporal dementia, or dementia with Lewy bodies. Sensitivity analyses using the weighted median method yielded consistent results, reinforcing the robustness of our findings. To uncover potential molecular mechanisms by which PA protects against cognitive decline, we analyzed transcriptomic data from the GSE110298 dataset in the Gene Expression Omnibus (GEO). Differential expression analysis revealed a distinct set of genes between patients with AD with high and low PA levels (Fig. 1C). Bayesian network modeling identified key pathways implicated in synaptic regulation, immune function, and neurodegenerative mechanisms (Fig. 1F). Gene Set Enrichment Analysis (GSEA) of differentially expressed genes (DEGs) showed significant enrichment in biological processes related to synaptic organization, neurogenesis, and synaptic signaling (Fig. 1D–E). Furthermore, a protein–protein interaction (PPI) network constructed from these DEGs highlighted hub genes such as *grin2b*, *gria2*, and *bdnf*, which play central roles in synaptic plasticity and neuronal resilience (Fig. 1G). Collectively, these multi-level findings suggest that PA improves cognitive performance, lowers the risk of AD, and promotes neuroplasticity and neurogenesis through the modulation of synaptic and molecular signaling pathways in individuals with AD.

### AE ameliorates cognitive deficits and Aβ pathology in APP/PS1 mice

**Fig. 2:**
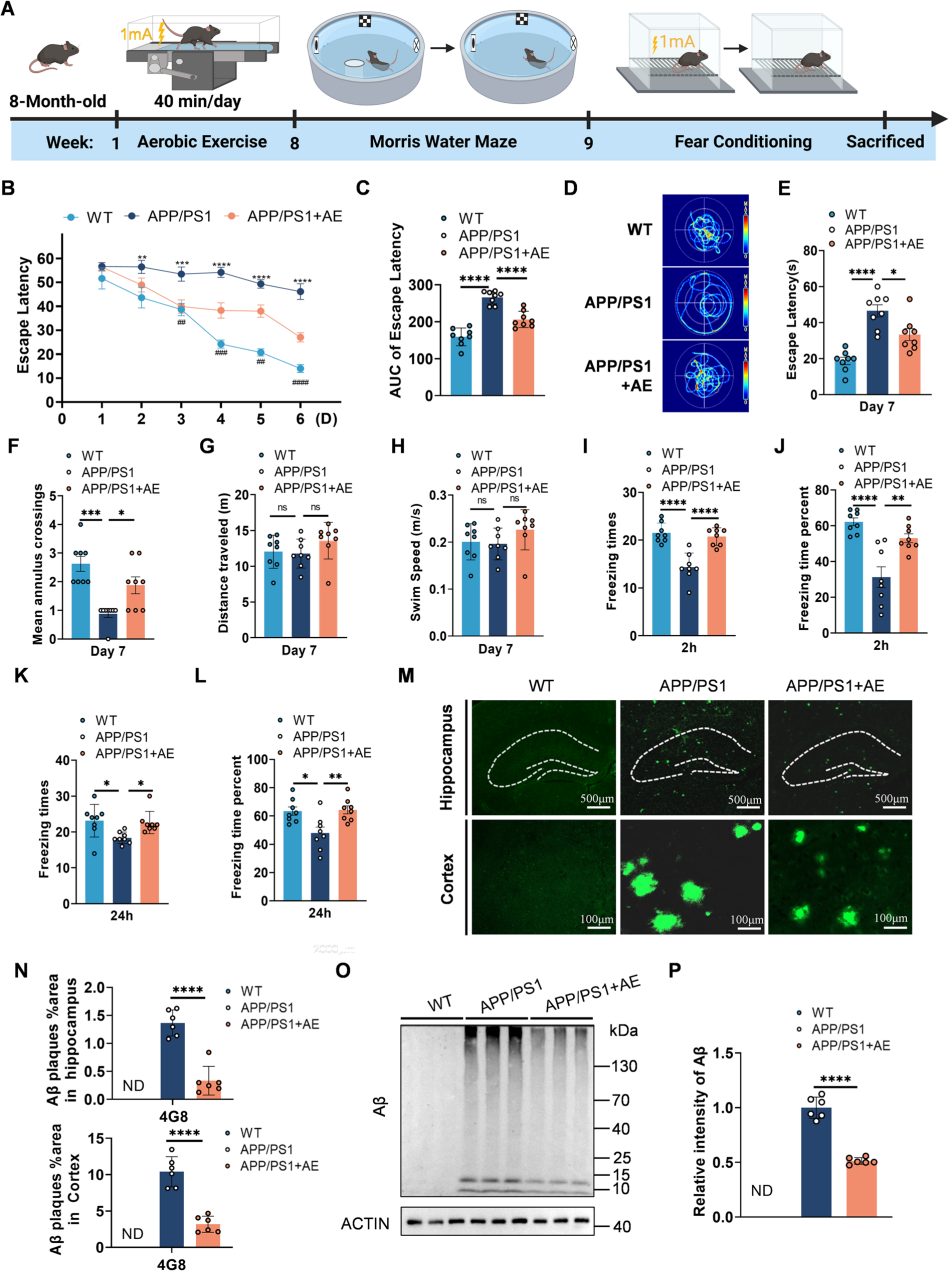
AE ameliorates cognitive deficits and Aβ pathology in APP/PS1 mice. **A** Schematic of the behavioral tests, including the Morris water maze (MWM) and contextual fear conditioning. **B** Escape latency during the 6-day acquisition training phase of the MWM (group × day interaction, F (10, 126) = 4.813, *p* < 0.0001 ). **C** Area under the curve (AUC) derived from the data in Fig. 1B (F(2, 21) = 49.98, *p* < 0.0001). **D** Representative swimming paths of mice from each group on day 7 (probe trial day). **E** Latency to first enter the target quadrant (F (2, 21) = 21.65, *p* < 0.0001) and (**F**) number of platform crossings during the probe trial (F (2, 21) = 13.45, *p* = 0.0002). **G** Total distance traveled (F (2, 21) = 1.428, *p* = 0.2621) and (**H**) average swimming speed of mice during the probe trial (F (2, 21) = 1.428, *p* = 0.2621). **I-J** Freezing times (F (2, 21) = 22.71, *p* < 0.0001) and freezing percentage (%) (F (2, 21) = 17.16, *p* < 0.0001) measured 2 h after contextual fear conditioning. **K-L** Freezing times (F (2, 21) = 4.963, *p* = 0.0172) and freezing percentage (%) (F (2, 21) = 7.363, *p* = 0.0038) measured 24 h after contextual fear conditioning. **M** Representative images of Aβ plaques detected by 4G8 immunostaining. **N** Quantification of the percentage of area occupied by Aβ plaques in the hippocampus (t = 7.315, df = 10, *p* < 0.0001) and cortex (t = 7.523, df = 10, *p* < 0.0001), respectively. **O** Western blot analysis of Aβ fractions extracted from the hippocampus. **P** Quantification of Aβ levels (t = 11.70, df = 10, *p* < 0.0001). Data are presented as mean ± SEM (*n* = 8 mice per group for behavioral tests; 3 mice per group with 2 sections each from the hippocampus and cortex for immunofluorescence; *n* = 6 mice per group for Western blot analysis). Two-way repeated-measures ANOVA followed by Bonferroni’s post hoc test was used for (B), while one-way ANOVA followed by Bonferroni’s post hoc test was used for (C–L), and Student’s t tests were used to analyze the data in (N-P). ^*^P < 0.05,^**^P < 0.01,^***^P < 0.001,^****^P < 0.0001 (WT vs. APP/PS1),^*#*^*P* < 0.05,^*##*^*P* < 0.01,^*###*^*P* < 0.001,^*####*^*P* < 0.0001 (APP/PS1 + AE vs. APP/PS1)

To validate the cognitive benefits of AE in an AD mouse model, APP/PS1 transgenic mice were subjected to an 8-week treadmill running protocol followed by behavioral assessments (Fig. 2A). During the Morris water maze (MWM) training phase, AE-treated APP/PS1 mice exhibited significantly shorter escape latencies compared with sedentary APP/PS1 controls from the 5th to 6th days (Fig. 2B). The area under the curve (AUC) analysis further confirmed improved learning performance in the AE group (Fig. 2C). During the probe test on day 7, AE mice exhibited more direct swim paths toward the previous platform location (Fig. 2D), decreased escape latency to the target quadrant (Fig. 2E), and an increased number of platform crossings (Fig. 2F), collectively indicating enhanced spatial memory retention. Total swimming distance and average velocity did not differ significantly between groups (Fig. 2G and H), excluding motor ability as a confounding factor. In the contextual fear conditioning test, AE significantly enhanced both recent (2-hour) and remote (24-hour) emotional associative memory, with increased freezing times (number of freezing events) and freezing time percentage compared with sedentary controls (Fig. 2I–L). Collectively, these results demonstrate that AE effectively ameliorates cognitive deficits in APP/PS1 mice. We further investigated the beneficial effects of AE on Aβ pathology in APP/PS1 mice. The immunofluorescence data showed that AE effectively alleviated Aβ accumulation (4G8) in the hippocampus and cortex of APP/PS1 mice (Fig. 2M-N). This reduction in Aβ load was further corroborated by western blot analysis of hippocampal lysates (Fig. 2O-P). Collectively, these findings indicate that AE not only improves cognitive performance but also alleviates Aβ pathology in APP/PS1 mice.

### AE modulates hippocampal neurons by reorganizing pseudotime trajectories and activating neuroactive ligand–receptor signaling and axon guidance pathways

**Fig. 3:**
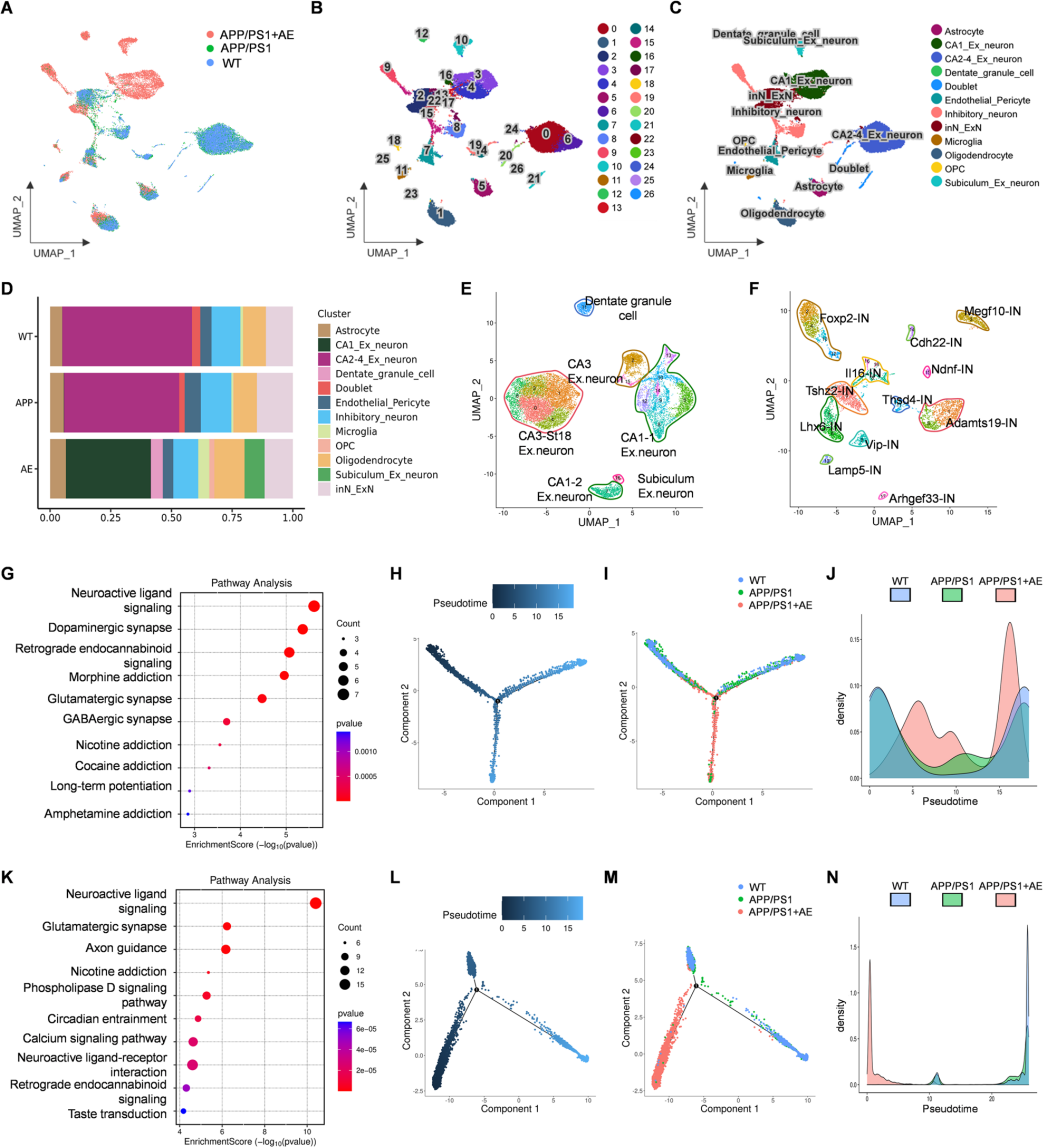
AE modulates hippocampal neurons by reorganizing pseudotime trajectories and activating neuroactive ligand–receptor signaling and axon guidance pathways. **A** Cellular distribution of hippocampal cells in WT, APP/PS1, and AE-treated APP/PS1 mice. **B** Clustering analysis of hippocampal cells from WT, APP/PS1, and AE-treated APP/PS1 mice. **C** Cell type annotation based on canonical marker genes, identifying major lineages. **D** Proportion of cell types in the hippocampus across WT, APP/PS1, and AE-treated APP/PS1 mice. **E-F** Subtyping and group-specific distribution of excitatory and inhibitory neuron populations in WT, APP/PS1, and AE-treated APP/PS1 mice. **G** KEGG pathway enrichment analysis of AE-upregulated genes in hippocampal inhibitory neurons. **H-J** Trajectory reconstruction and pseudotime distribution of inhibitory neurons across WT, APP/PS1, and AE-treated APP/PS1 mice. **K** KEGG pathway enrichment analysis of AE-upregulated genes in hippocampal excitatory neurons. **L-N** Trajectory reconstruction and pseudo-time distribution of excitatory neurons across WT, APP/PS1, and AE-treated APP/PS1 mice

To investigate the cellular effects of AE in AD, we performed single-cell RNA sequencing (scRNA-seq) on hippocampal tissues from wild-type (WT), APP/PS1, and AE-treated APP/PS1 mice (Fig. 3A). Unbiased clustering analysis revealed distinct cellular populations across the three groups (Fig. 3B). Cell type annotation, based on canonical marker genes, identified major hippocampal lineages, including excitatory neurons, inhibitory neurons, astrocytes, microglia, oligodendrocytes, and vascular cells (Fig. 3C). A comparative analysis of hippocampal cellular composition revealed distinct cell type distributions among the three groups (Fig. 3D). Further sub-clustering and subsequent analyses of excitatory and inhibitory neurons showed group-specific distribution changes (Fig. 3E–F). Functional enrichment analysis of AE-upregulated genes revealed that both inhibitory and excitatory neurons were primarily associated with neuroactive ligand–receptor signaling, axon guidance, and other neurofunction-related pathways (Fig. 3G, K). Pseudotime trajectory analysis revealed distinct distribution patterns of different neuronal subtypes among the three groups. For inhibitory neurons, cells from the WT and APP/PS1 groups were mainly located at the initial and terminal segments of the differentiation trajectory. In contrast, AE induced a shift in cell distribution toward the central portion of the trajectory, indicating a relatively homogeneous and stable state (Fig. 3H–J). In excitatory neurons, the APP/PS1 group exhibited a heterogeneous and branched trajectory structure, while AE-treated cells were tightly aligned along a single trajectory branch and predominantly located at the root of the trajectory. This suggests an early activation state and potential functional plasticity (Fig. 3L–N). Collectively, these findings suggest that AE restores hippocampal homeostasis by reorganizing neuronal trajectories and enhancing neuroprotective signaling pathways in APP/PS1 mice.

### AE restores synaptic protein expression and plasticity in APP/PS1 mice

**Fig. 4:**
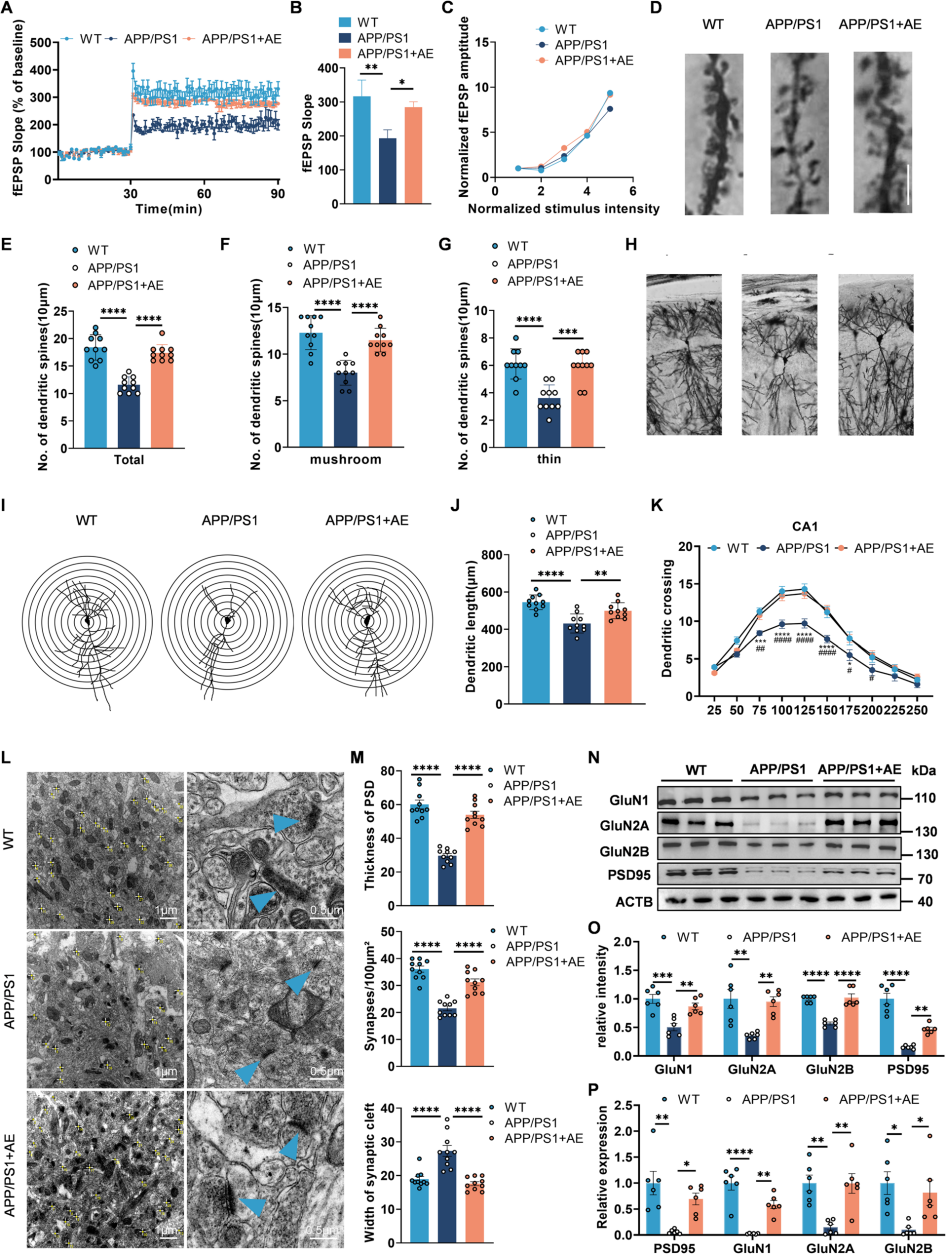
Aerobic exercise ameliorates synaptic plasticity impairments in APP/PS1 mice. **A–B** Field excitatory postsynaptic potential (fEPSP) slopes recorded from the CA1 region (F (2, 6) = 11.73, *p* = 0.0084). **C** Input-output curve detected from CA1 region. **D** Representative images of dendrites from hippocampal CA1 neurons via Golgi staining (scale bar: 5 μm). **E** Quantification of total dendritic spine density (F (2, 27) = 41.74, *p* < 0.0001). **F, G** Density of mushroom-shaped (F (2, 27) = 23.30, *p* < 0.0001) and thin dendritic spines (F (2, 27) = 17.25, *p* < 0.0001), respectively (*n* = 10 neurons from 3 mice each group, scale bar = 5 μm). **H–I** Representative images of hippocampal CA1 neurons with Golgi staining. **J-K** Determination of the dendritic length (F (2, 27) = 16.22, *p* < 0.0001) and sholl analysis of dendritic crossing point amounts in CA1 neurons (group × dendritic crossing interaction, F (18, 270) = 2.926, *p* < 0.0001). **L** Representative transmission electron microscopy (TEM) images of synapses in the hippocampal CA1 region (*n* = 10 images from 2 mice each group, scale bar = 1 μm). **M** Quantitative analysis of synaptic density (F (2, 27) = 45.59, *p* < 0.0001), synaptic cleft width (F (2, 27) = 23.48, *p* < 0.0001), and postsynaptic density (PSD) thickness (F (2, 27) = 59.87, *p* < 0.0001) from TEM images (*n* = 10 synapses per group from 5 images, scale bar = 0.5 μm). **N–O** Protein expression levels of key synaptic markers (PSD95, F (2, 15) = 49.64, *p* < 0.0001; GluN1, F (2, 15) = 14.66, *p* = 0.0003; GluN2A, F (2, 15) = 11.05, *p* = 0.0011; GluN2B, F (2, 15) = 34.96, *p* < 0.0001) were analyzed by Western blot in WT, APP/PS1, and APP/PS1 + AE mice (*n* = 6 per group). **P** The mRNA levels of PSD95 (F (2, 15) = 10.75, *p* = 0.0013), GluN1 (F (2, 15) = 29.74, *p* < 0.0001), GluN2A (F (2, 15) = 11.43, *p* = 0.0010), and GluN2B (F (2, 15) = 6.169, *p* = 0.0111) were tested by RT‒PCR (*n* = 6 per group). Data are presented as mean ± SEM. One-way ANOVA followed by Bonferroni’s post hoc test was used to analyze the data in (B–J, M-P). Two-way ANOVA followed by Bonferroni’s post hoc test was used to analyze the data in (K). *P < 0.05, **P < 0.01, ***P < 0.001, ****P < 0.0001 (WT vs. APP/PS1), #*P* < 0.05, ##*P* < 0.01, ###*P* < 0.001, ####*P* < 0.0001 (APP/PS1 + AE vs. the APP/PS1)

To evaluate the protective effect of AE on synaptic plasticity in AD, we performed a comprehensive analysis of synaptic function in the hippocampus of APP/PS1 mice. Electrophysiological recordings at the Schaffer collateral–CA1 pathway revealed a significant impairment in long-term potentiation (LTP) in APP/PS1 mice, as reflected by reduced fEPSP slopes compared to WT controls (Fig. 4A–B). AE intervention robustly enhanced LTP in APP/PS1 mice, bringing fEPSP responses back to levels comparable to those of WT animals (Fig. 4A–B). In contrast, paired-pulse facilitation remained unchanged across groups, indicating that presynaptic function remained unaltered (Fig. 4C). Collectively, these results demonstrate that AE effectively rescues deficits in hippocampal synaptic plasticity in APP/PS1 mice, likely through a postsynaptic mechanism. Golgi staining further showed a marked reduction in dendritic spine density in CA1 hippocampal neurons of APP/PS1 mice compared to WT controls (Fig. 4D). AE significantly elevated spine density in CA1 neurons, including both mushroom-shaped and thin spines (Fig. 4E–G), suggesting enhanced synaptic stability. Additionally, Sholl analysis (radius interval: 250 μm) revealed that APP/PS1 mice exhibited reduced dendritic branching complexity and shorter total dendritic length. AE increased the number of dendritic crossings, branch points, and total length in CA1 neurons (Fig. 4H–K). Transmission electron microscopy (TEM) uncovered severe synaptic ultrastructural deficits in APP/PS1 mice, including reduced synapse density (per 100 μm²), widened synaptic clefts, and thinner postsynaptic densities (PSD) compared with WT controls (Fig. 4L–M). AE intervention substantially mitigated these deficits, restoring synaptic density, narrowing synaptic cleft width, and increasing PSD thickness (Fig. 4L–M), indicative of synaptic ultrastructure recovery. Western blot analysis showed that the expression of key synaptic proteins (PSD95, GluN1, GluN2A, GluN2B) was significantly reduced in APP/PS1 mice (Fig. 4N). AE treatment rescued these proteins to levels comparable to those in WT mice (Fig. 4O). Quantitative RT-PCR confirmed AE-induced upregulation of synaptic genes at the mRNA level (Fig. 4P). Collectively, these results demonstrate that AE restores synaptic density and ultrastructure, enhances dendritic complexity, and rescues synaptic protein expression in APP/PS1 mice, thereby improving synaptic plasticity and potentially counteracting neurodegeneration.

### AE enhances histone acetylation and restores synaptic gene expression in APP/PS1 mice

**Fig. 5:**
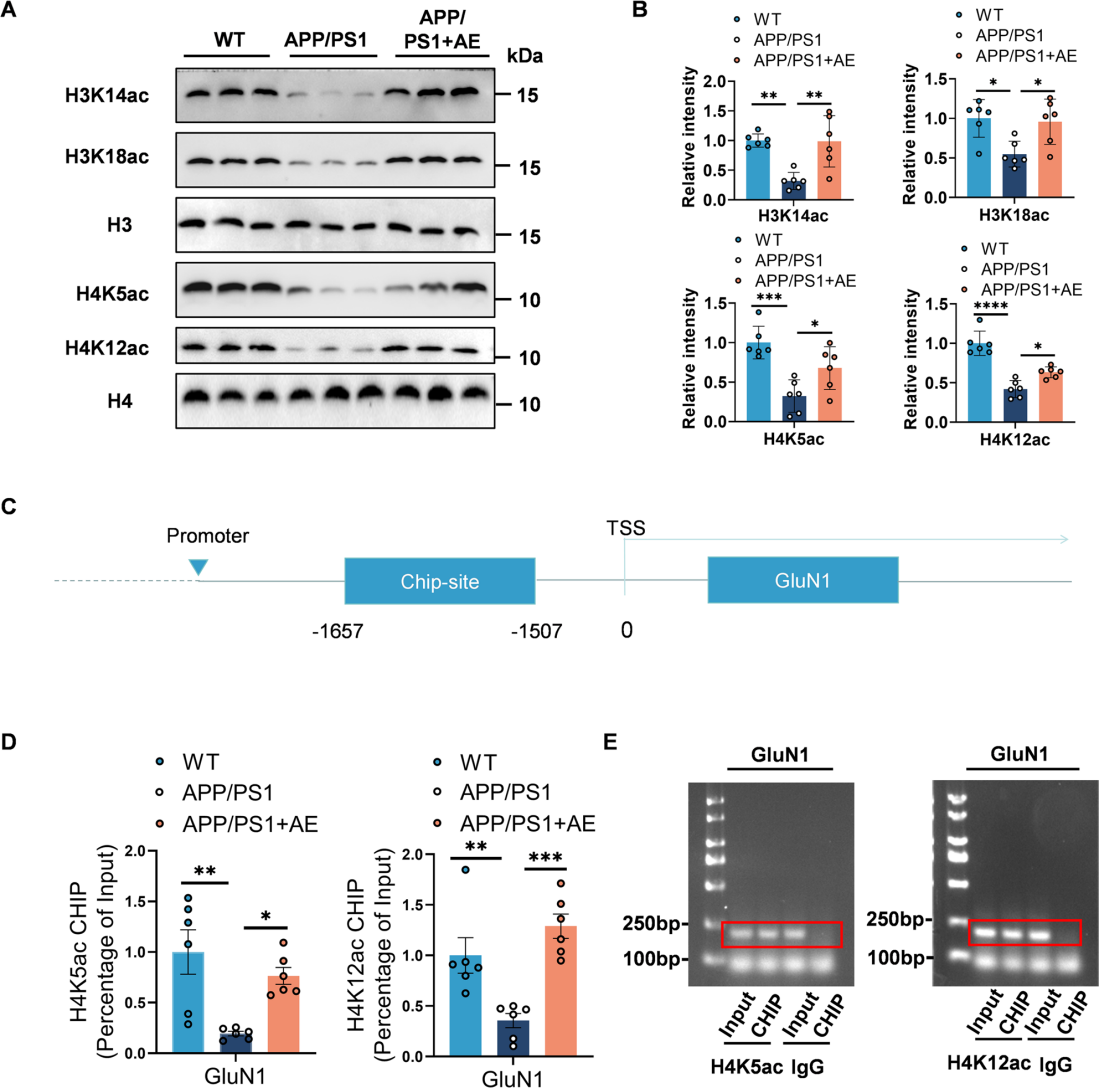
Aerobic exercise enhances histone acetylation at the GluN1 promoter in APP/PS1 mice. **A-B** Western blot analysis of global histone acetylation levels (H3K14ac, F (2, 15) = 12.38, *p* = 0.0007; H3K18ac, F (2, 15) = 6.775, *p* = 0.0080; H4K5ac, F (2, 15) = 13.06, *p* = 0.0005; H4K12ac, F (2, 15) = 38.58, *p* < 0.0001) in hippocampal tissues (*n* = 6 per group). **C** Schematic of the mouse GluN1 (Grin1) promoter region, indicating the putative binding sites for H4K5 and H4K12 acetylation. **D-E** Chromatin immunoprecipitation followed by qPCR (ChIP-qPCR) quantifying the enrichment of H4K5ac (F (2, 15) = 9.299, *p* = 0.0024) and H4K12ac (F (2, 15) = 13.72, *p* = 0.0004) at the GluN1 promoter (*n* = 6 per group). Data are presented as mean ± SEM. Statistical significance was determined by one-way ANOVA followed by Bonferroni’s post hoc test. *P < 0.05; **P < 0.01; ***P < 0.001; ****P < 0.0001

Histone acetylation plays a pivotal role in synaptogenesis and synaptic plasticity by modulating the transcription of synapse-related genes [55]. To investigate the epigenetic effects of AE in AD, we analyzed hippocampal histone acetylation in the hippocampus of APP/PS1 mice. Western blot analysis showed significantly reduced acetylation of H3K14, H3K18, H4K5, and H4K12 (p300/CBP substrate targets [56] and reduced in AD [57]) in APP/PS1 mice compared with WT controls (Fig. 5A–B). AE intervention substantially restored the acetylation of these histone residues (Fig. 5A–B), indicating AE-mediated chromatin remodeling. To determine whether AE modulates histone acetylation specifically at synaptic gene promoters, we performed chromatin immunoprecipitation coupled with quantitative PCR (ChIP-qPCR) targeting the promoter region of GluN1 (–1,657 to − 1,507 bp relative to the transcription start site, TSS; Fig. 5C). We observed a marked reduction of H4K5ac and H4K12ac 157enrichment at the GluN1 promoter in the hippocampus of APP/PS1 mice. AE treatment effectively rescued these histone acetylation deficits (Fig. 5D). Agarose gel analysis of ChIP-PCR amplicons further confirmed AE-induced acetylation changes (e.g., H4K12ac at the GluN1 promoter; Fig. 5E). These findings demonstrate that AE restores synaptic gene expression in APP/PS1 mice by enhancing histone acetylation at key promoters, providing a mechanistic link to improved synaptic plasticity and cognition.

### AE induces ADRB2-AMPK signaling-dependent nuclear translocation of p300 in APP/PS1 mice

**Fig. 6:**
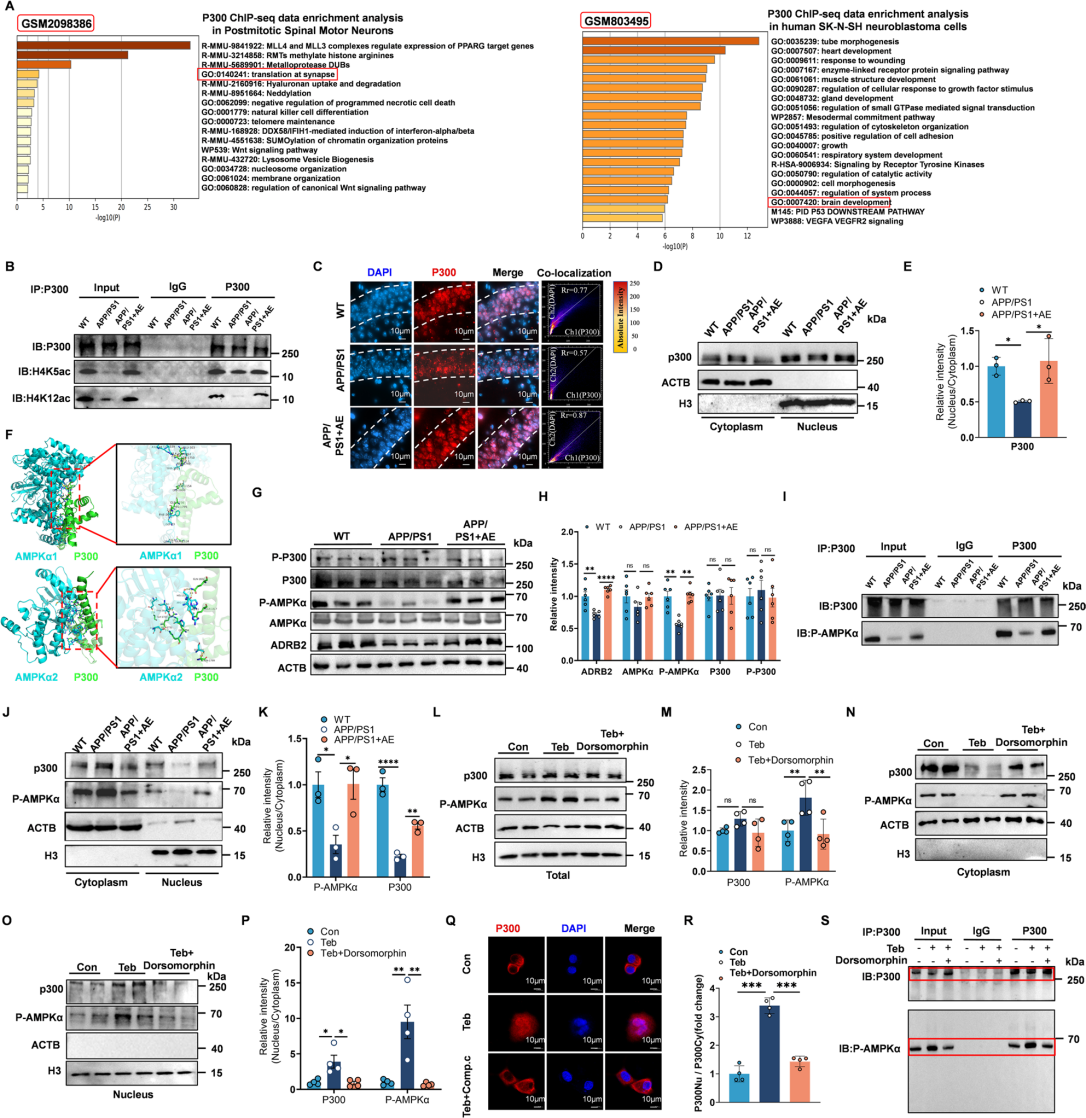
Aerobic exercise activates ADRB2 signaling and promotes nuclear translocation of p300 and phosphorylated AMPKα in APP/PS1 mice. **A** Metascape enrichment analysis of p300 ChIP-seq data from the top 200 target genes (Dataset: GSM2098386) and top 300 target genes (Dataset: GSM803495). **B** Co-immunoprecipitation (Co-IP) analysis of interactions among p300, H4K5ac, and H4K12ac. **C** Representative immunofluorescence images showing the subcellular distribution of p300 in hippocampal sections from WT, APP/PS1, and AE-treated APP/PS1 mice. **D–E **Western blot and quantitative analysis (F (2, 6) = 7.475, *p* = 0.0235) of cytoplasmic and nuclear fractions of p300 in hippocampal tissues (*n* = 3 per group). **F** Molecular docking simulation showing the interaction between p300 and AMPKα (AMPKα1 and AMPKα2). **G** Western blot analysis of total protein levels of p300, P-p300, AMPKα, p-AMPKα, and ADRB2 in hippocampal tissues from WT, APP/PS1, and APP/PS1 + AE mice (*n* = 6 per group). **H** Quantification of protein expression levels of p300 (F (2, 15) = 0.003039, *p* = 0.9970), P-p300 (F (2, 15) = 0.1982, *p* = 0.8223), AMPKα (F (2, 15) = 1.304, *p* = 0.3004), p-AMPKα (F (2, 15) = 20.42, *p* < 0.0001), and ADRB2 (F (2, 15) = 18.62, *p* < 0.0001) across groups. **I** Co-IP analysis of interactions among p300, and p-AMPKα. **J-K** Subcellular fractionation showing nuclear and cytoplasmic distribution of p300 (F (2, 6) = 60.24, *p* = 0.0001) and p-AMPKα (F (2, 6) = 7.487, *p* = 0.0234). **L-P** Western blot analysis of total (p300, F (2, 9) = 2.754, *p* = 0.1166; p-AMPKα, F (2, 9) = 7.507, *p* = 0.0121), nuclear and cytoplasmic fractions (p-AMPKα, F (2, 9) = 13.28, *p* = 0.0021; p300, F (2, 9) = 9.404, *p* = 0.0062) of p300 and p-AMPKα in N2A cells (*n* = 4 per group). **Q** Immunofluorescence images showing nuclear accumulation of p300 in N2A cells treated with Terbutaline (Teb) or Terbutaline plus AMPK inhibitor (Dorsomorphin) (*n* = 4 per group). **R** Quantification of nuclear p300 fluorescence intensity across treatment conditions (F (2, 9) = 103.3, *p* < 0.0001). **S** Co-IP confirming the interaction between p300 and p-AMPKα in N2A cells under different treatments. Data are presented as means ± SEMs. One-way ANOVA followed by Bonferroni’s post hoc test was used for analysis of (E, H, K, M, P, R). *P < 0.05; **P < 0.01; ***P < 0.001; ****P < 0.0001

The HAT family, particularly p300/CBP in the nervous system, plays a critical role in modulating chromatin remodeling at genes associated with synaptic plasticity and memory formation [58]. To investigate the potential involvement of p300 in exercise-induced epigenetic regulation, we performed pathway enrichment analysis using p300 ChIP-seq data obtained from mouse spinal motor neurons (GSM2098386) and retinoic acid-differentiated human SK-N-SH neuroblastoma cells (GSM803495). This analysis indicated that genes marked by p300 occupancy were significantly enriched in transcriptional networks that govern neurodevelopment and synaptic function (Fig. 6A). Building on these findings, we examined whether p300 contributes to the AE-mediated enhancement of histone acetylation. Co-immunoprecipitation (Co-IP) assays demonstrated that AE strengthened the interaction between p300 and histones acetylated at H4K5 and H4K12 (Fig. 6B). To elucidate the mechanism through which AE modulates p300 to promote histone acetylation, we next examined the expression and subcellular localization of p300 in mouse brain tissue. Western blot analysis indicated no significant alteration in total p300 protein levels. In contrast, immunofluorescence staining revealed a notable alteration in its subcellular distribution. In hippocampal sections from APP/PS1 mice, p300 was primarily cytoplasmic, with substantially reduced nuclear presence. However, AE treatment induced a significant increase in the nuclear accumulation of p300 in the hippocampus of APP/PS1 mice (Fig. 6C). Nuclear fractionation assays further quantitatively confirmed that AE promotes p300 nuclear translocation (Fig. 6D–E). Recent studies indicate that p300 nucleocytoplasmic shuttling underlies AMPK signaling, and disruption of this regulatory axis is closely implicated in neuropathological processes such as aberrant mTORC1 activation [59, 60]. Molecular docking (hdock) demonstrated direct p300-AMPKα1/2 binding (Fig. 6F). To investigate whether AMPK is also involved in the regulation and mechanism of AE-mediated p300 nuclear translocation, we first examined changes in AMPK protein expression and activity. Western blot analysis showed that AE increased the level of AMPK phosphorylated at Thr172 (p-AMPK) without affecting total AMPK expression (Fig. 6G–H). Co-IP further demonstrated that the interaction between p300 and p-AMPK was reduced in the hippocampus of APP/PS1 mice, and this interaction was significantly enhanced by AE (Fig. 6I). This suggests that AMPK binding may regulate the subcellular localization of p300. To test this hypothesis, we performed cytoplasmic and nuclear fractionation of mouse hippocampal tissues. Both p-AMPK and p300 exhibited similar distribution patterns, and AE promoted nuclear accumulation of each (Fig. 6J–K). To further investigate whether p-AMPK is a key mediator in the nuclear import of p300, we treated Neuro-2a (N2a) cells with terbutaline (Teb), an ADRB2 agonist, to mimic the effects of AE due to the fact that our previous research indicated that AE activates autophagy through the ADRB2-AMPK pathway. We conducted these experiments both in the presence and absence of the AMPK inhibitor dorsomorphin, and subsequently analyzed the subcellular localization of p300. Western blot results indicated that Teb treatment had no significant effect on total p300 protein levels but increased p-AMPK levels, an effect that was attenuated by dorsomorphin (Fig. 6L–M). cellular fractionation assays revealed that Teb treatment induced a pan-cellular distribution of p300, while dorsomorphin inhibited its nuclear translocation (Fig. 6N–P). This finding was further corroborated by Immunofluorescence staining (Fig. 6Q–R). Furthermore, Co-IP results confirmed that Teb enhanced the binding between p-AMPK and p300, whereas dorsomorphin suppressed this interaction (Fig. 6S). Taken together, these data indicate that AE activates the ADRB2-AMPK pathway, leading to AMPK-dependent nuclear translocation of p300. Subsequently, nuclear p300 enhances histone acetylation and promotes the expression of learning- and memory-associated genes.

### The N-terminal 200 amino acids of p300 mediate AMPKα binding and are essential for nuclear localization

**Fig. 7:**
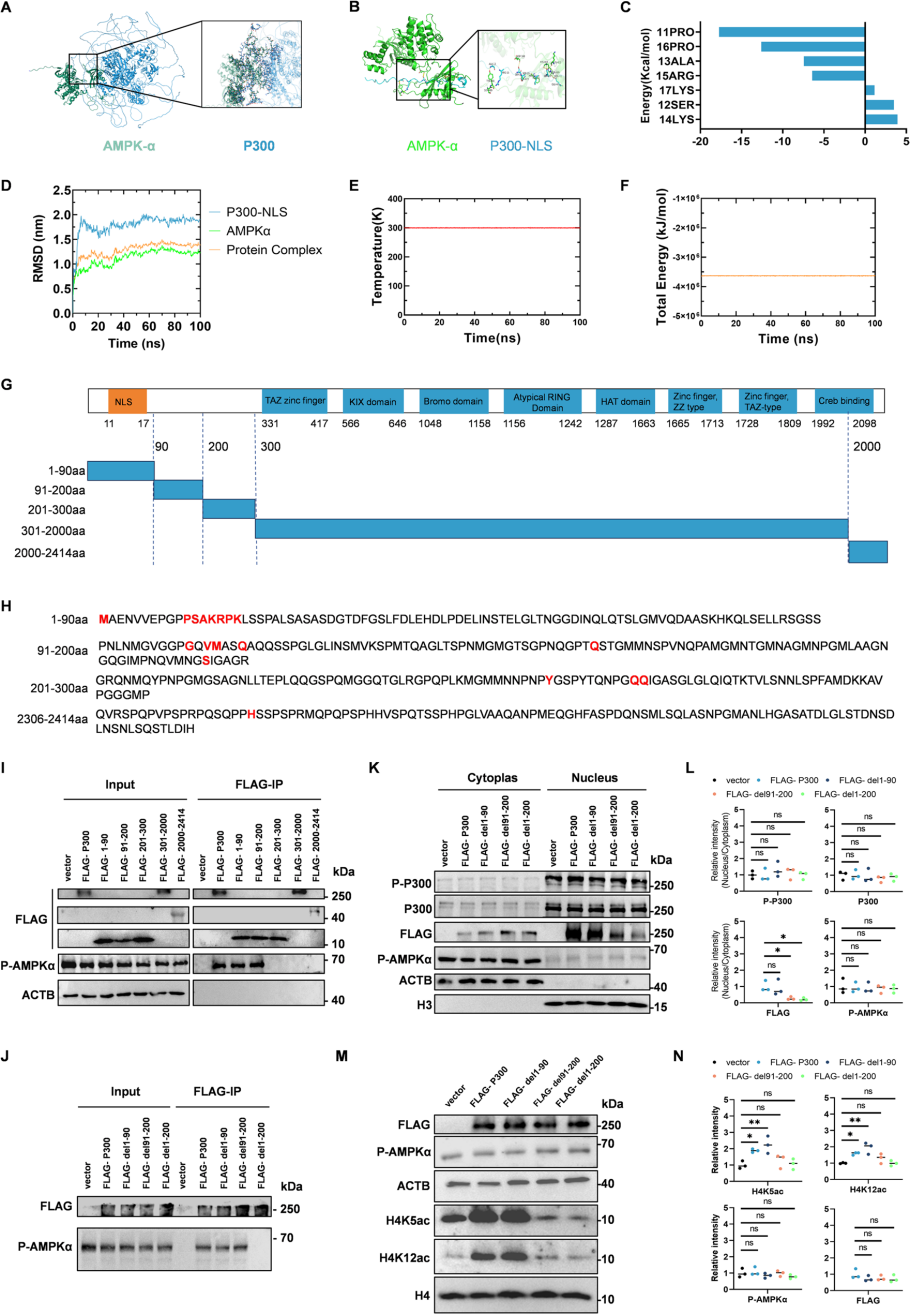
The N-terminal 200 Amino Acids of p300 Are Required for AMPKα Binding and Nuclear Localization. **A** Molecular docking model depicting the binding between full-length p300 and AMPKα. **B, C** The energy contributions of each residue of p300-NLS interaction with AMPKα. **D** Root-mean-square deviation (RMSD) of the p300-NLS/AMPKα complex throughout a 100 ns molecular dynamics simulation, reflecting structural stability. **E** Temperature monitoring during the molecular dynamics simulation process. **F** Total system energy variations during simulation. **G** Schematic diagram of FLAG-tagged p300 truncation constructs. **H** Predicted binding interfaces between p300 fragments and p-AMPKα from molecular docking. **I, J** Co-IP analysis showing that p300 1–90 aa and 91–200 aa fragments bind p-AMPKα, while deletion of both regions abolishes binding. **K-L** Subcellular fractionation analysis showing the relative nuclear/cytoplasmic intensity of P300 (F (4, 10) = 0.3520, *p* = 0.8370), P-P300 (F (4, 10) = 0.5094, *p* = 0.7305), P-AMPKα (F (4, 10) = 0.09604, *p* = 0.9814), and p300 mutants (F (3, 8) = 5.616, *p* = 0.0228) after terbutaline treatment. **M-N** Western blot analysis of histone acetylation markers (H4K5ac, F (4, 10) = 6.946, *p* = 0.0061; H4K12ac, F (4, 10) = 9.694, *p* = 0.0018), P-AMPKα (F (4, 10) = 1.154, *p* = 0.3867), and p300 mutants (F (3, 8) = 1.078, *p* = 0.4115) in cells expressing different p300 constructs. Data are presented as means ± SEMs. One-way ANOVA followed by Bonferroni’s post hoc test was used for analysis of (L, N). *P < 0.05; **P < 0.01; ***P < 0.001; ****P < 0.0001

To further validate the interaction between p300 and AMPK and identify the specific binding region, we employed molecular docking to predict the binding between full-length p300 and AMPKα (Fig. 7A). Subsequent analysis using a p300 truncation mutant (amino acids 1–40) containing the nuclear localization sequence (NLS, residues 11–17) revealed that NLS residues directly interact with AMPKα (Fig. 7B). Energy contribution analysis further confirmed a strong binding affinity between individual residues within the p300-NLS and AMPKα (Fig. 7C). Molecular dynamics simulations over 100 ns demonstrated the structural stability of the p300-NLS/AMPKα complex, as reflected by root mean square deviation (RMSD) analysis. Consistent with this, both temperature and total energy profiles showed minimal fluctuations, confirming the stability of the simulated system (Fig. 7D–F). To experimentally map the AMPKα-binding region, we constructed a series of Flag-tagged p300 truncation mutants (1–90 aa, 91–200 aa, 201–300 aa, 301–2000 aa, and 2001–2414 aa) (Fig. 7G). Residues predicted by docking analysis to contact AMPKα are highlighted in red (Fig. 7H). Co-IP assays confirmed that both the 1–90 aa and 91–200 aa fragments of p300 bind to p-AMPKα (Fig. 7I). Deletion of both regions of p300 completely abolished the interaction with p-AMPKα (Fig. 7J).

To investigate the functional role of the AMPKα-p300 binding region in p300 nuclear import and histone acetylation, HEK293 cells expressing different p300 fragments were treated with Teb. Results showed that deletion of either the 91–200 aa or 1–200 aa region significantly impaired the nuclear accumulation of Flag-p300 (Fig. 7K-L). Interestingly, histone acetylation analysis revealed that while deletion of the 1–90 aa region had no significant effect, loss of the 91–200 aa region markedly reduced the acetylation levels of H4K5 and H4K12 (Fig. 7M-N). These findings suggest that although the 1–200 aa segment of p300 mediates its nuclear translocation, the 91–200 aa region is critical for its histone acetyltransferase activity targeting H4K5 and H4K12.

### Dorsomorphin abolishes the beneficial effects of AE on histone acetylation upregulation and cognitive improvement in APP/PS1 mice

**Fig. 8:**
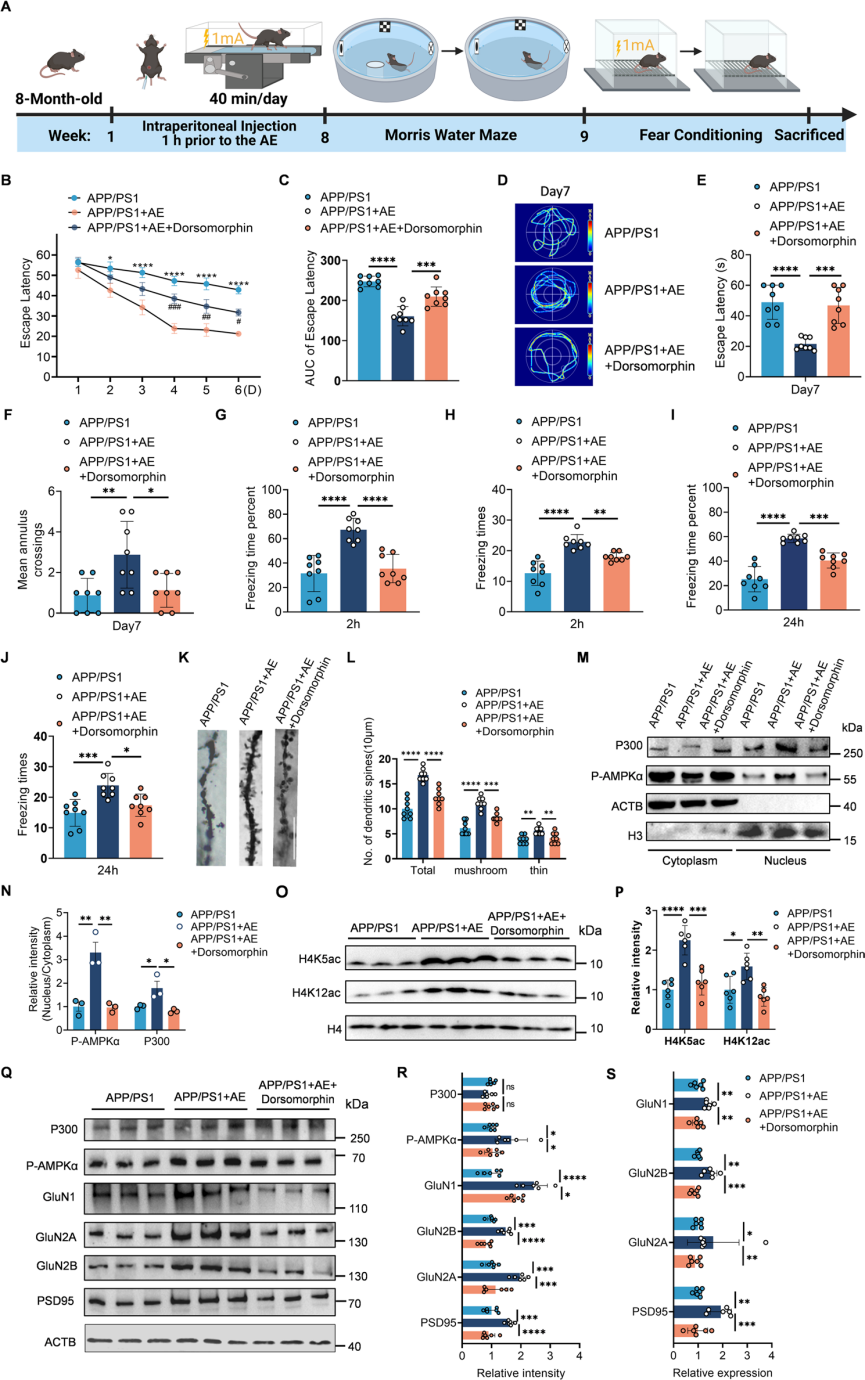
AMPK inhibition with Dorsomorphin abolishes the cognitive and synaptic benefits of aerobic exercise in APP/PS1 mice. **A** Experimental timeline of behavioral tests, including the Morris water maze and contextual fear conditioning, in APP/PS1, APP/PS1 + AE, and APP/PS1 + AE+Dorsomorphin mice. **B** Escape latency during the 6-day training phase of the Morris water maze (group × day interaction, F F (10, 126) = 2.103, *p* = 0.0288). **C** Area under the curve (AUC) of escape latency during training phase (F (2, 21) = 34.19, *p* < 0.0001). **D-F** Probe trial analysis assessing swimming path, first latency to platform (F (2, 21) = 19.73, *p* < 0.0001), and number of platform crossings (F (2, 21) = 6.969, *p* = 0.0048). **G–J** Fear conditioning test results evaluating freezing times and freezing time percentage at 2-hour (freezing times, F (2, 21) = 26.48, *p* < 0.0001; freezing time percentage, F (2, 21) = 20.89, *p* < 0.0001 and 24-hour (freezing times, F (2, 21) = 10.80, *p* = 0.0006; freezing time percentage, F (2, 21) = 42.11, *p* < 0.0001) intervals post-conditioning. **K** Representative images of Golgi-stained dendrites from hippocampal CA1 neurons (scale bar = 5 μm). **L** Quantification of dendritic spine density in CA1 neurons (total dendritic spine density, F (2, 21) = 36.90, *p* < 0.0001; mushroom-shaped spine density, F (2, 21) = 36.17, *p* < 0.0001; thin spine density, F (2, 21) = 8.539, *p* = 0.0019; *n* = 8 neurons from 2 mice each group). **M, N** Subcellular fractionation showing nuclear localization of p300 (F (2, 6) = 8.501, *p* = 0.0177) and p-AMPKα (F (2, 6) = 22.28, *p* = 0.0017) in hippocampal tissues (*n* = 3 per group). **O, P** Western blot analysis of histone acetylation markers H4K5ac (F (2, 15) = 26.73, *p* < 0.0001) and H4K12ac (F (2, 15) = 10.20, *p* = 0.0016) in hippocampal tissues (*n* = 6 per group). **Q, R** Western blot analysis of synaptic proteins (GluN1, F (2, 15) = 26.39, *p* < 0.0001; GluN2A, F (2, 15) = 18.24, *p* < 0.0001; GluN2B, F (2, 15) = 25.87, *p* < 0.0001; PSD95, F (2, 15) = 25.02, *p* < 0.0001), p300 (F (2, 15) = 0.9910, *p* = 0.3942), and p-AMPKα (F (2, 15) = 6.533, *p* = 0.0091) (*n* = 6 per group). **S** The mRNA levels of glun1 (F (2, 15) = 11.04, *p* = 0.0011), glun2a (F (2, 15) = 7.522, *p* = 0.0055), glun2b (F (2, 15) = 18.13, *p* < 0.0001) and psd95 (F (2, 15) = 14.02, *p* = 0.0004) were tested by RT‒PCR (*n* = 6 per group). Data are presented as means ± SEMs. Two-way ANOVA followed by Bonferroni’s post hoc test was used for analysis of (B). One-way ANOVA followed by Bonferroni’s post hoc test was used for analysis of (C–S). *P < 0.05, **P < 0.01, ***P < 0.001, ****P < 0.0001 (APP/PS1 + AE vs. APP/PS1), #*P* < 0.05, ##*P* < 0.01, ###*P* < 0.001, ####*P* < 0.0001 (APP/PS1 + AE+Dorsomorphin vs. APP/PS1 + AE). Proposed working model for aerobic exercise

To investigate whether AMPKα signaling is essential for the beneficial effects of AE on the nuclear translocation of p300 and synaptic plasticity in AD mice, we treated AE-exposed APP/PS1 mice with dorsomorphin (an AMPK inhibitor, 10 mg/kg, intraperitoneal injection) (Fig. 8A). In the MWM test, dorsomorphin treatment impaired the learning performance of AE mice from days 4 to 6, as reflected by increased escape latency and a higher area under the curve (AUC) of escape latency (Fig. 8B, C). During the probe trial on day 7, dorsomorphin-treated AE mice exhibited longer swimming paths, longer escape latencies, and significantly fewer platform crossings (Fig. 8D–F). In the fear conditioning test, AE significantly improved both recent (2-hour) and remote (24-hour) emotional associative memory in APP/PS1 mice, as evidenced by increased freezing times and freezing time percentage compared to sedentary controls (Fig. 8G–J). However, the administration of dorsomorphin attenuated these improvements, leading to reduced freezing times and freezing time percentage during both memory phases (Fig. 8G–J). Golgi staining revealed that AE increased dendritic spine density in hippocampal CA1 neurons, an effect that was reversed by AMPK inhibition (Fig. 8K–L). Subcellular fractionation analysis revealed that AE promoted the nuclear localization of p300 and p-AMPKα, while dorsomorphin treatment significantly reduced this localization (Fig. 8M–N). At the epigenetic level, AE increased the acetylation of histone marks H4K5 and H4K12, suggesting increased chromatin accessibility, while dorsomorphin reversed these effects (Fig. 8O–P). At the synaptic level, Western blot and qPCR analyses demonstrated that AE restored the expression of synaptic proteins GluN1, GluN2A, GluN2B, and PSD95 at both protein and mRNA levels, whereas dorsomorphin markedly suppressed these improvements (Fig. 8Q–S). Collectively, these findings indicate that dorsomorphin can negate the beneficial effects of AE on histone acetylation and cognitive improvement by inhibiting the p-AMPKα-p300 axis in APP/PS1 mice.

## Discussion

This study elucidates a previously unrecognized mechanism by which AE exerts neuroprotective effects against AD via a metabolism-epigenetics regulatory (ADRB2-AMPK-p300) axis. We demonstrate that AE activates the ADRB2, promotes the phosphorylation of AMPK, and ultimately drives the nuclear translocation of the histone acetyltransferase p300. This process enhances histone H4K5 and H4K12 acetylation in an AMPK signaling-dependent manner, leading to the transcriptional activation of synaptic-related genes such as PSD95, GluN1, GluN2A, and GluN2B, thereby significantly improving learning and memory performance in APP/PS1 transgenic mice. Furthermore, we found that the N-terminal (1–200 aa) domain of p300 is critical for its interaction with p-AMPKα, nuclear import, and histone acetylation, expanding our understanding of p300’s regulatory mechanisms. These findings define a novel signal transduction pathway through which behavioral interventions (e.g., exercise) are translated into chromatin remodeling, offering a promising epigenetic target for AD therapy.

Previous studies have elucidated the relationship between exercise dose and outcomes such as cancer survival and depression [3, 61]. The nonlinear dose-response relationship between PA and cognitive performance, identified in the NHANES cohort, provides a crucial epidemiological foundation; it not only confirms the association in a representative human population but also defines the optimal activity levels that maximize cognitive benefit, thereby informing the intensity and design of our animal exercise interventions. Previous studies have suggested that strenuous sports or other exercises can reduce the risk of Tourette’s syndrome (TS) [62]. Our MR analysis extends beyond correlation to suggest a causal protective effect of genetically proxied PA specifically against AD. This genetic evidence significantly strengthens the biological plausibility of our hypothesis and justifies a focused investigation into AD-related mechanisms in the animal model rather than a general exploration of cognitive function. While exercise as a beneficial intervention is well established, our study advances the field by delineating the specific neurobiological pathways, including synaptic regulation [63], neurogenesis [64], and immune modulation [65], through which these effects are mediated, as highlighted by our subsequent transcriptomic and network analyses (GSE110298). Thus, the human data are not peripheral but integral to our work, providing the causative, dose-responsive, and disease-specific context that guides and validates the mechanistic discoveries from our animal experiments.

The role of physical exercise in mitigating aging-related cognitive decline and preventing neurodegenerative diseases is an area of growing interest in public health and neurobiological research [66]. AE such as running and swimming have been consistently associated with preserved cognitive function in aging populations, likely through mechanisms involving vascular health, neuroinflammation reduction, and enhanced neurotrophic signaling [67, 68]. Resistance training, including bodyweight exercises, contributes to improved metabolic profiles, notably increased muscle mass, insulin sensitivity, and IGF-1 secretion, which may secondarily support neuronal health; though direct evidence concerning its effects on cognitive outcomes remains limited [69, 70]. Mind–body practices (e.g., Tai Chi, Yoga) appear to modulate autonomic balance and stress response pathways, thereby attenuating chronic inflammation and offering potentially distinct benefits for affective regulation and neuroprotection [71, 72]. Notably, sex-specific differences in exercise-induced neuroadaptations have emerged, possibly mediated by gonadal hormones [73]. Estrogen may augment the neuroprotective effects of AE in females, particularly through synaptic plasticity mechanisms [74]; testosterone, conversely, could influence resistance training-induced neural adaptations in males [75]. While each exercise modality engages distinct physiological pathways, their combinatorial effects warrant further mechanistic investigation. Ultimately, tailored exercise interventions hold promise as strategic approaches for delaying cognitive aging and reducing the burden of neurodegenerative disorders [75].

Numerous studies have demonstrated that AE confers significant benefits in alleviating AD pathology and cognitive impairment. Epidemiological data show that regular moderate-intensity exercise significantly reduces the risk of developing AD and delays disease progression [76, 77]. At the mechanistic level, AE enhances neurogenesis, upregulates brain-derived neurotrophic factor (BDNF), improves mitochondrial function, promotes synaptic remodeling, and reduces neuroinflammation [78–82]. However, how these peripheral and short-term metabolic changes induced by AE are translated into sustained transcriptional adaptations in the brain remains unclear. In particular, the chromatin-level mechanisms through which AE activates neuroprotective transcriptional programs are poorly understood. Clarifying this signaling gap could both explain long-term cognitive effects of AE and provide non-physical alternatives for individuals with limited mobility due to AD.

Accumulating evidence suggests that epigenetic dysregulation centered on histone acetylation is a key driver of AD progression. In both patients and animal models of AD, acetylation levels at sites such as H3K9, H3K18, and H4K12 are significantly reduced, leading to the repression of critical synaptic plasticity genes, including BDNF, Arc, and Egr1 [83–86]. Impaired expression or activity of HATs such as p300/CBP has been implicated as a major contributor [87]. Therapeutic approaches aiming to restore histone acetylation, such as HDAC inhibitors or HAT activators, have shown improvements in learning and memory across various AD models [88–90]. p300 is not only a critical histone acetyltransferase but also a transcriptional co-activator involved in the assembly of transcriptional complexes and promoter regulation. p300 exhibits condition-dependent nuclear localization, with its translocation regulated by metabolic stress and cofactors such as BAT3 and β-arrestin1, thereby determining its epigenetic activity [60, 91–93]. We discovered that the N-terminal 1–200 aa domain of p300 contains both the nuclear localization signal (NLS) and the interface required for binding p-AMPKα, making it essential for AE-induced nuclear entry. This structure-dependent mechanism ensures that p300 is properly mobilized by exercise-induced metabolic signals to enact histone acetylation. Nuclear accumulation of p300 is thought to enhance the formation of transcriptional complexes with factors such as CREB, CBP, and MED1, facilitating the coordinated activation of neuroprotective genes. This mechanism may underlie a molecular basis for the long-term memory enhancement seen with exercise, a form of “epigenetic memory.” Notably, although deletion of the 91–200 aa region did not disrupt the interaction between p300 and p-AMPKα, it significantly attenuated p300 nuclear translocation and its downstream histone acetylation, indicating that this segment may contribute to the enzymatic function of p300 or be subject to regulation by additional nuclear transport factors.

ADRB2 and AMPK serve as key metabolic sensors that regulate cellular metabolism and function, mitochondrial activity, and inflammation [94–97]. ADRB2 activation enhances AMPK–PKA signaling, promotes mitochondrial function, and supports neuronal survival [98, 99]; AMPK, as an energy sensor, regulates mitochondrial biogenesis and autophagy and modulates transcription through phosphorylation of downstream targets [100, 101]. Moreover, p-AMPKα translocates into the nucleus, where it interacts with transcriptional and epigenetic regulators to orchestrate gene expression programs related to metabolism [102, 103]. Our results show that AE upregulates ADRB2-AMPKα-p300 signaling, which drives the nuclear translocation of p300, enhances histone acetylation, and mitigates cognitive decline in APP/PS1 mice. Pharmacological inhibition of AMPK (e.g., with dorsomorphin) blocks this signaling cascade, confirming that AE activates the p300 epigenetic program via an ADRB2-AMPK-dependent mechanism. This work fills a critical knowledge gap by establishing a direct mechanistic link between metabolic signaling and chromatin remodeling, providing a comprehensive explanation of how AE modulates cognitive function at the molecular level.

### Limitations and future directions

First, all experiments were performed in APP/PS1 transgenic mice, a familial AD model that does not fully represent the complexity and heterogeneity of sporadic AD, thereby limiting the translational relevance of the findings. Furthermore, the exercise intervention relied solely on a treadmill-running paradigm, without evaluating alternative exercise types, intensities, or durations, which restricts the optimization of exercise strategies. In addition, sex differences were not assessed, even though sex hormones may modulate ADRB2 and AMPK signaling and thus could influence the neuroprotective effects of exercise. Moreover, although single-cell analyses were included, the study did not employ spatially resolved epigenomic approaches, leaving the spatial distribution and temporal progression of p300 nuclear translocation and associated chromatin remodeling events insufficiently characterized. Lastly, while dorsomorphin has been documented in the literature to target the ATP-binding site of the catalytic α subunit of AMPK [104], it is indeed known to exhibit non-specific inhibitory effects, including potential impacts on the BMP signaling pathway and other kinases. Such non-specificity represents a pharmacological limitation in this study. Nevertheless, our conclusion that AMPKα activity is necessary remains supported by the observation that dorsomorphin consistently disrupts the complete sequential phenotype triggered by exercise, spanning from initial AMPK activation to downstream epigenetic and cognitive outcomes. This coherent disruption accords with established AMPK‑dependent mechanisms and contrasts with typical effects of BMP inhibition. Future studies employing more selective AMPKα inhibitors would help to further solidify these mechanistic insights. Looking forward, future studies should validate the ADRB2–AMPKα–p300 axis in sporadic AD models and human brain tissue, compare diverse exercise modalities to establish optimized interventions, and perform sex-stratified analyses to define sex-dependent responses. The development of small-molecule activators that mimic exercise-induced epigenetic effects may further provide personalized or exercise-mimetic therapeutic options for AD.

## Conclusion

Through integrated epidemiological, multi-omics, pathological, and molecular approaches, we demonstrate that AE significantly reduces AD risk and improves cognitive function in AD models. Mechanistically, we identify the ADRB2-AMPKα-p300 signaling axis as critical for these neuroprotective effects: AE activates ADRB2, inducing AMPKα phosphorylation and subsequent nuclear translocation of p300 via its N-terminal 1–200 aa domain binding with p-AMPKα. This cascade drives histone hyperacetylation (H4K5ac/H4K12ac) at synaptic gene promoters (GluN1), restoring neuroprotective gene expression and synaptic plasticity. The necessity of this pathway is confirmed by the complete blockade of AE benefits upon AMPKα inhibition (dorsomorphin). Our findings not only bridge metabolic signaling to epigenetic remodeling but also reveal actionable therapeutic targets for developing exercise-mimetic therapies, particularly for mobility-limited patients with AD.

## Supplementary Information

Supplementary Material 1.

## Data Availability

Data is provided within the manuscript.

## Abbreviations

PA: Physical activity
AD: Alzheimer's disease
Aβ: Amyloid-β
AE: Aerobic exercise
ADRB2: β2-adrenergic receptor
GPCR: G protein-coupled receptor
cAMP: Cyclic AMP
AMPK: AMP-activated protein kinase
LTP: Long-term potentiation
CHIP: Chromatin immunoprecipitation
Co-IP: Co-immunoprecipitation
MWM: Morris water maze
GluN2B: N-methyl-D-aspartate receptor type 2B
GluN1: N-methyl-D-aspartate receptor 1
GluN2A: N-methyl-D-aspartate receptor type 2A
PDS95: Postsynaptic Density-95
NHANES: National Health and Nutrition Examination Survey
MR: Mendelian randomization
GEO: Gene Expression Omnibus
ANOVA: Analysis of variance
SEM: Standard error of the mean
