# Supplementary material for: Aerobic exercise facilitates p300 nuclear translocation via ADRB2-AMPKα signaling, leading to enhanced histone acetylation and mitigation of cognitive decline in APP/PS1 mice
Source: Alzheimers Res Ther. 2026 Feb 10;18:62. doi: 10.1186/s13195-026-01983-z (PMC12990585; doi:10.1186/s13195-026-01983-z)

Source Data for Figure 2O

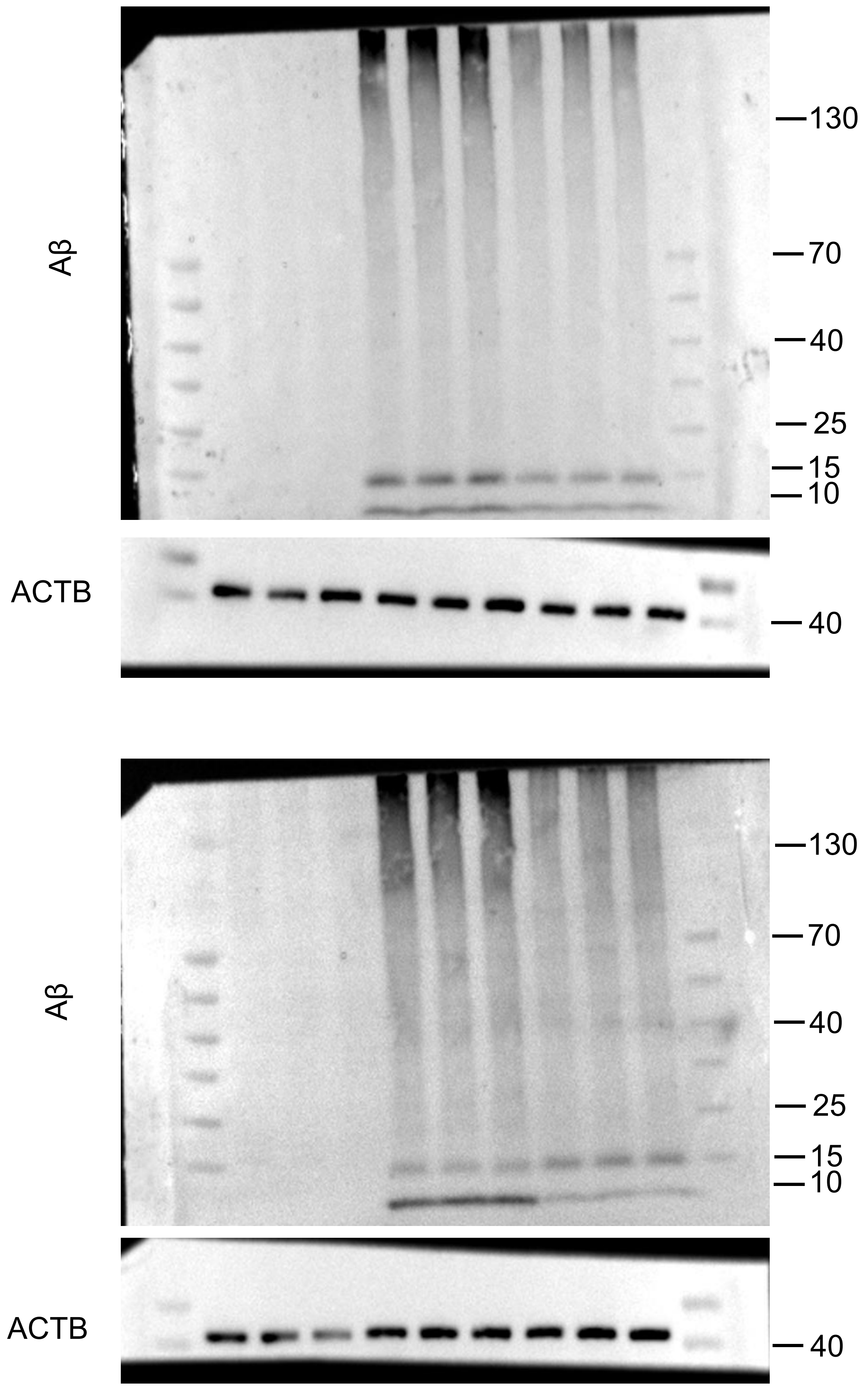

Source Data for Figure 4N

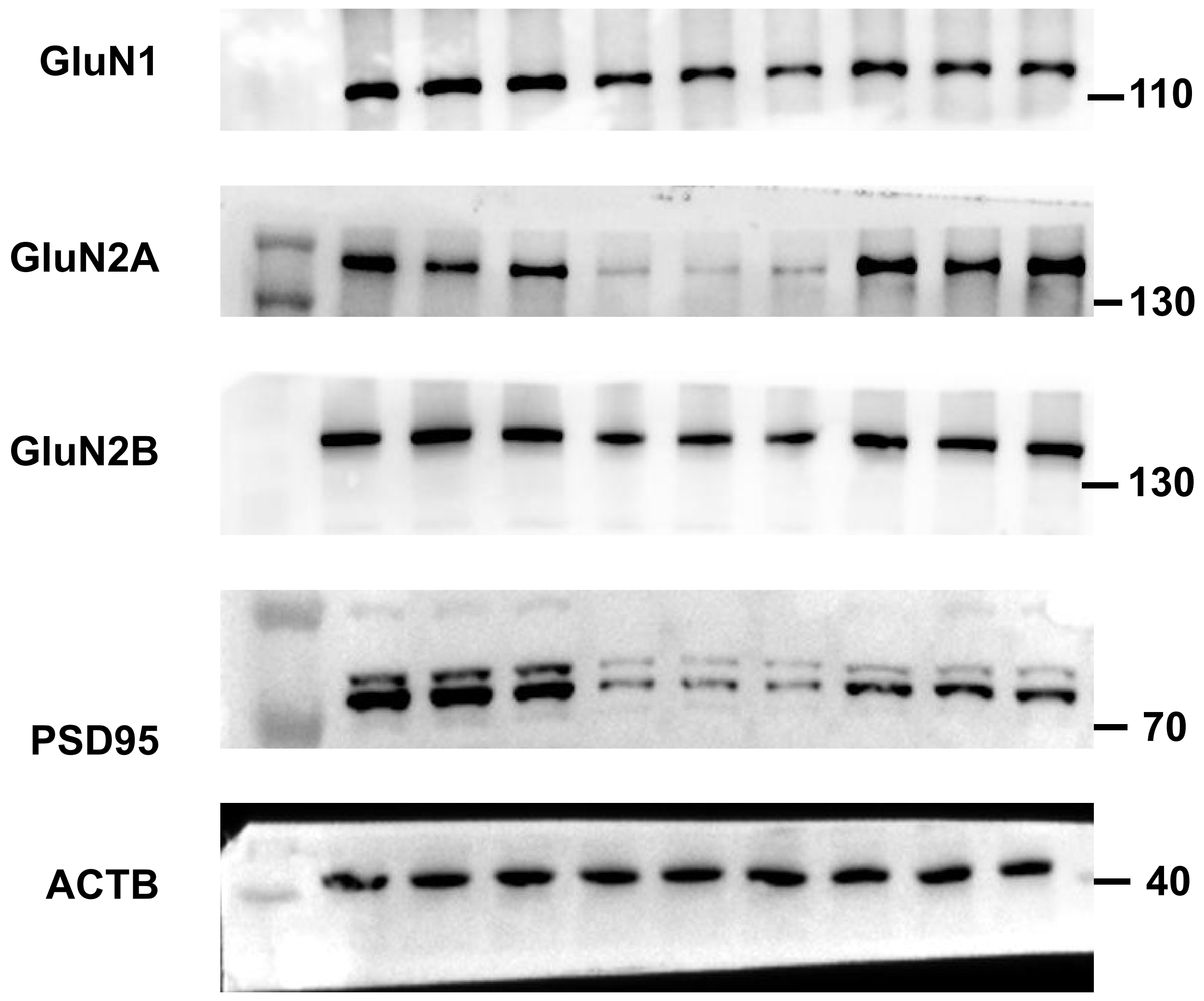

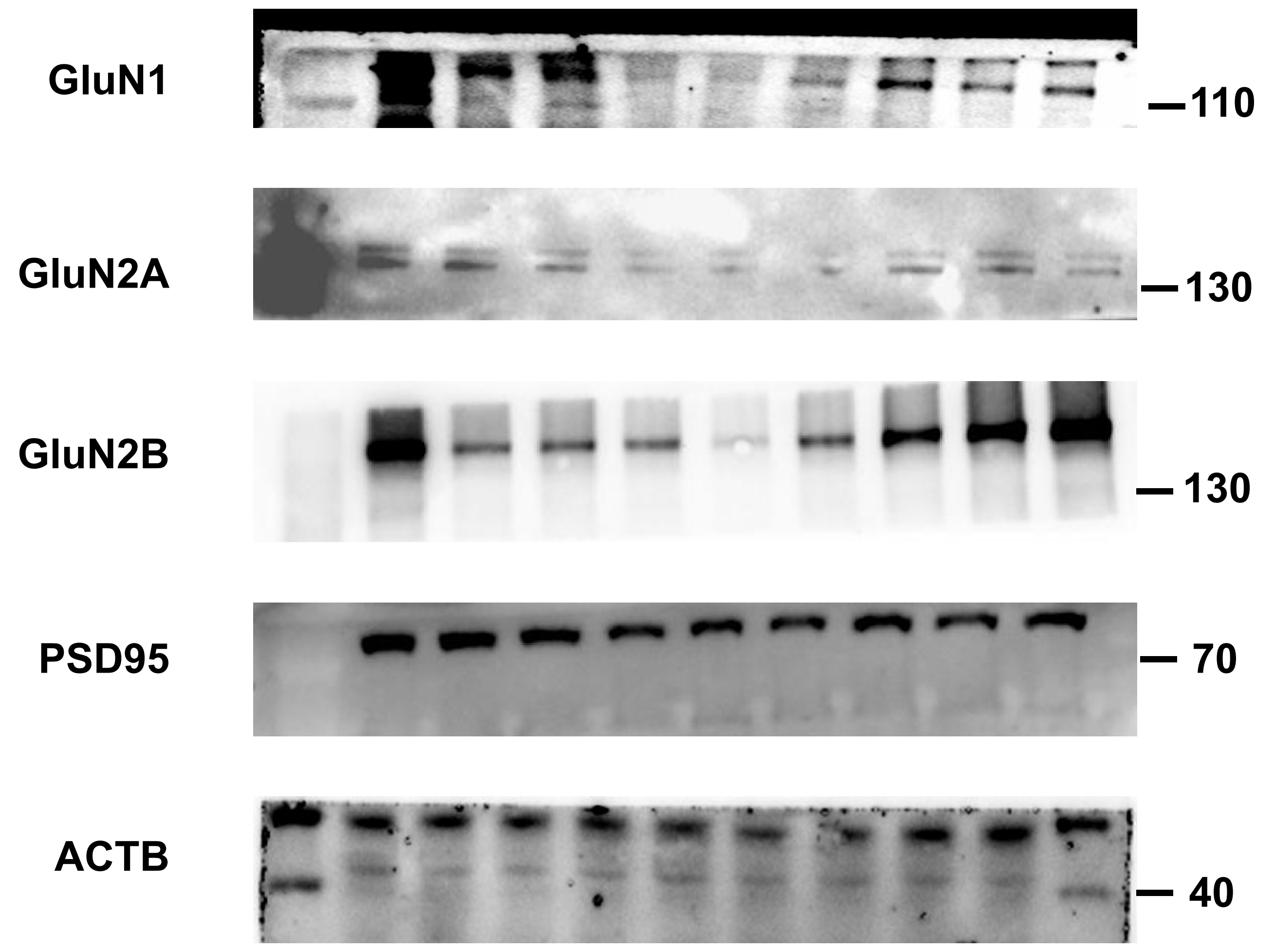

Source Data for Figure 5A

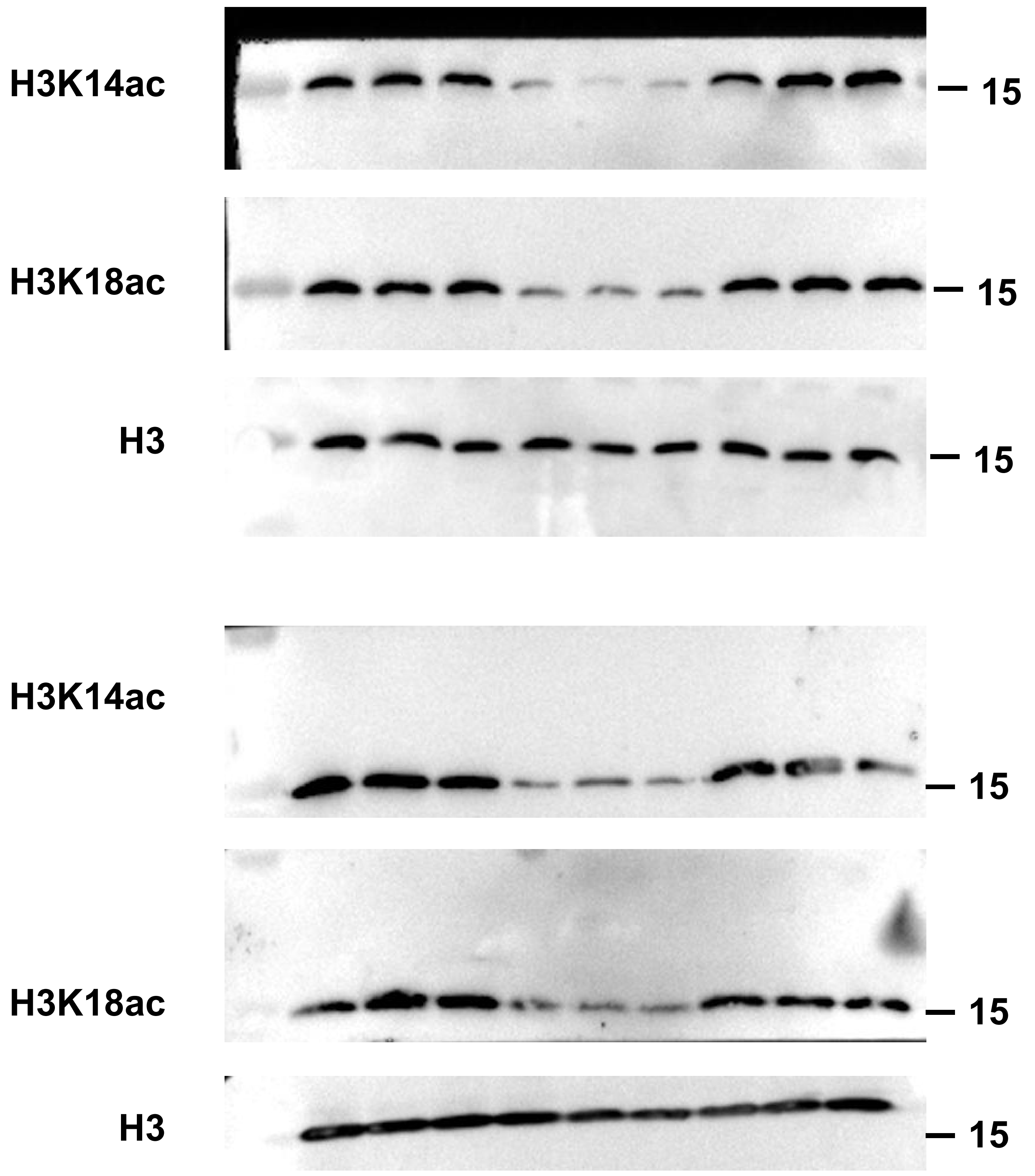

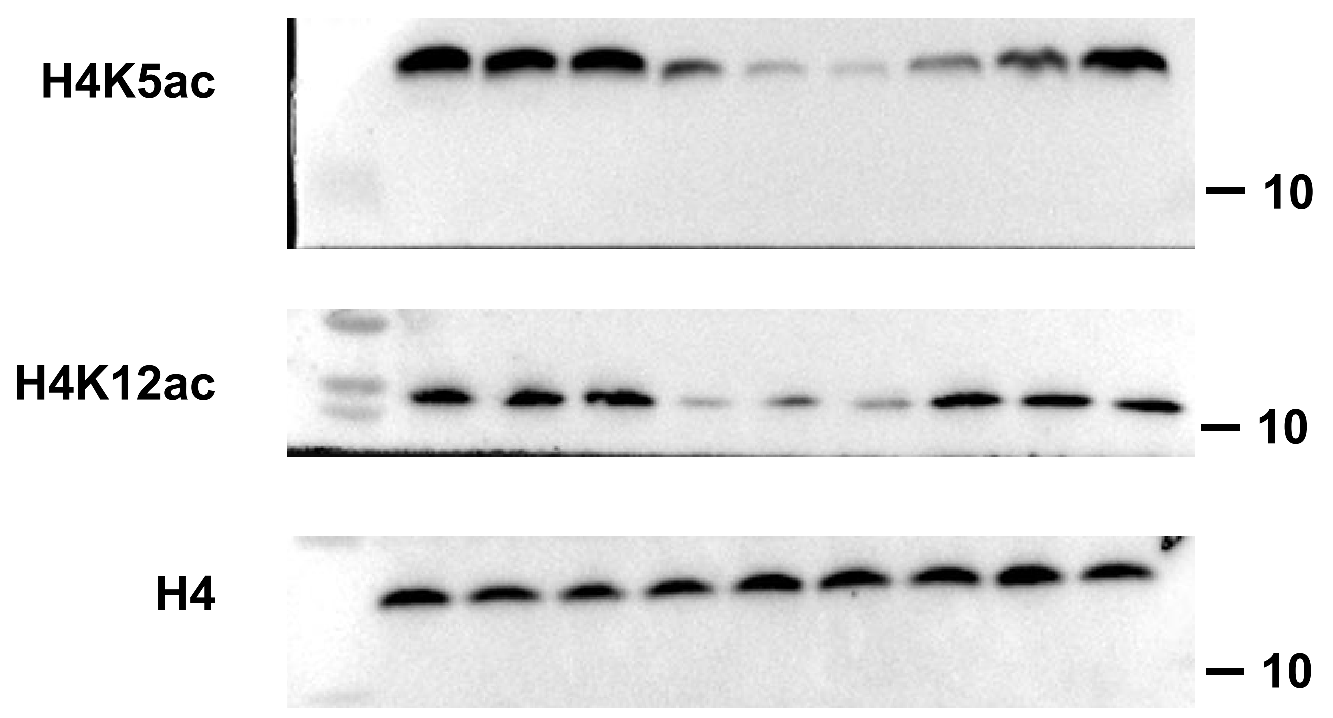

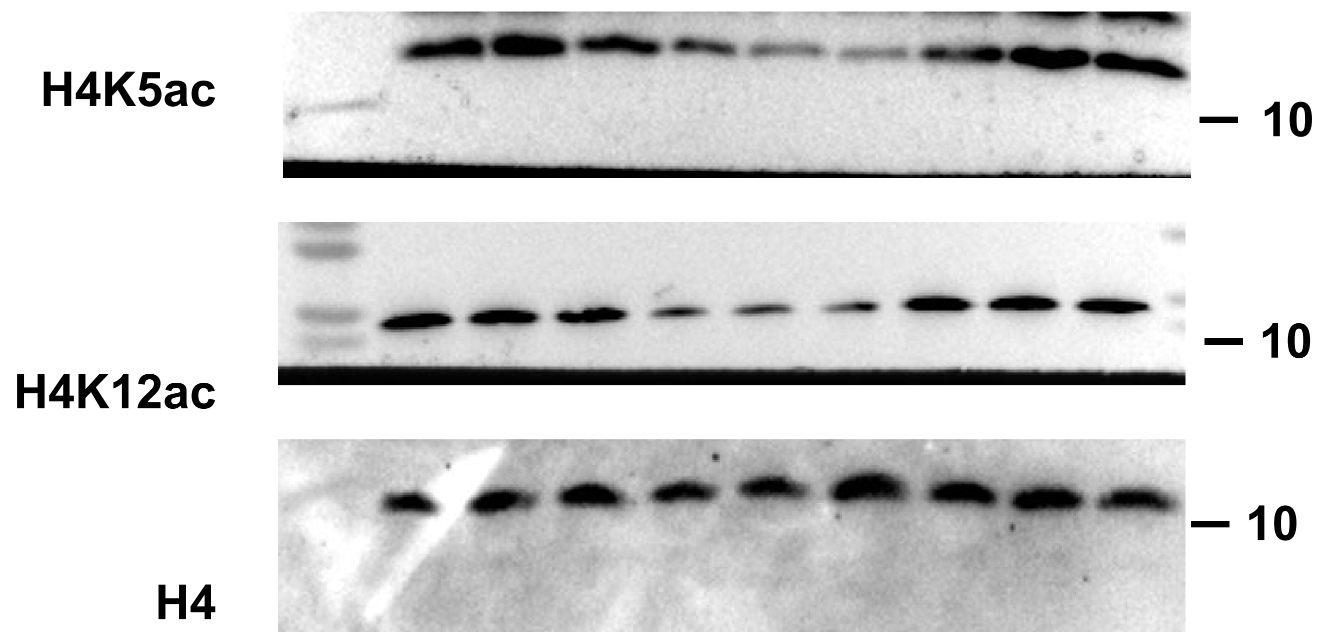

Source Data for Figure 6B

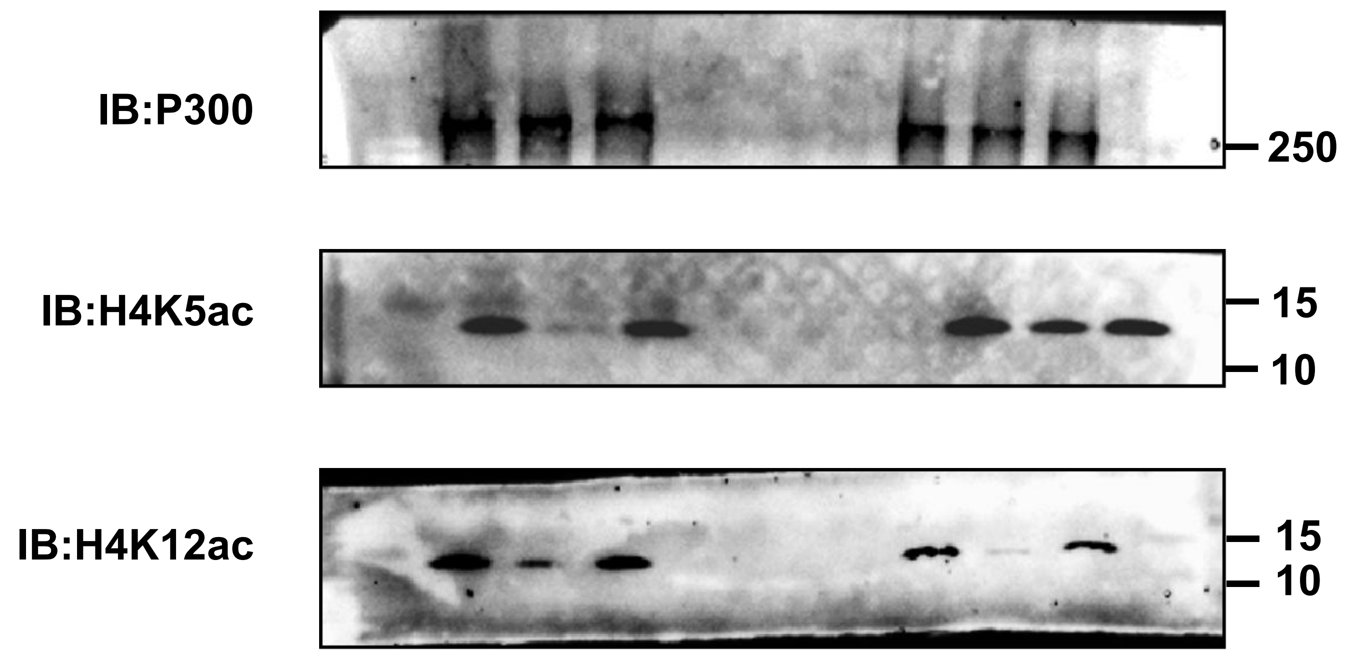

Source Data for Figure 6D

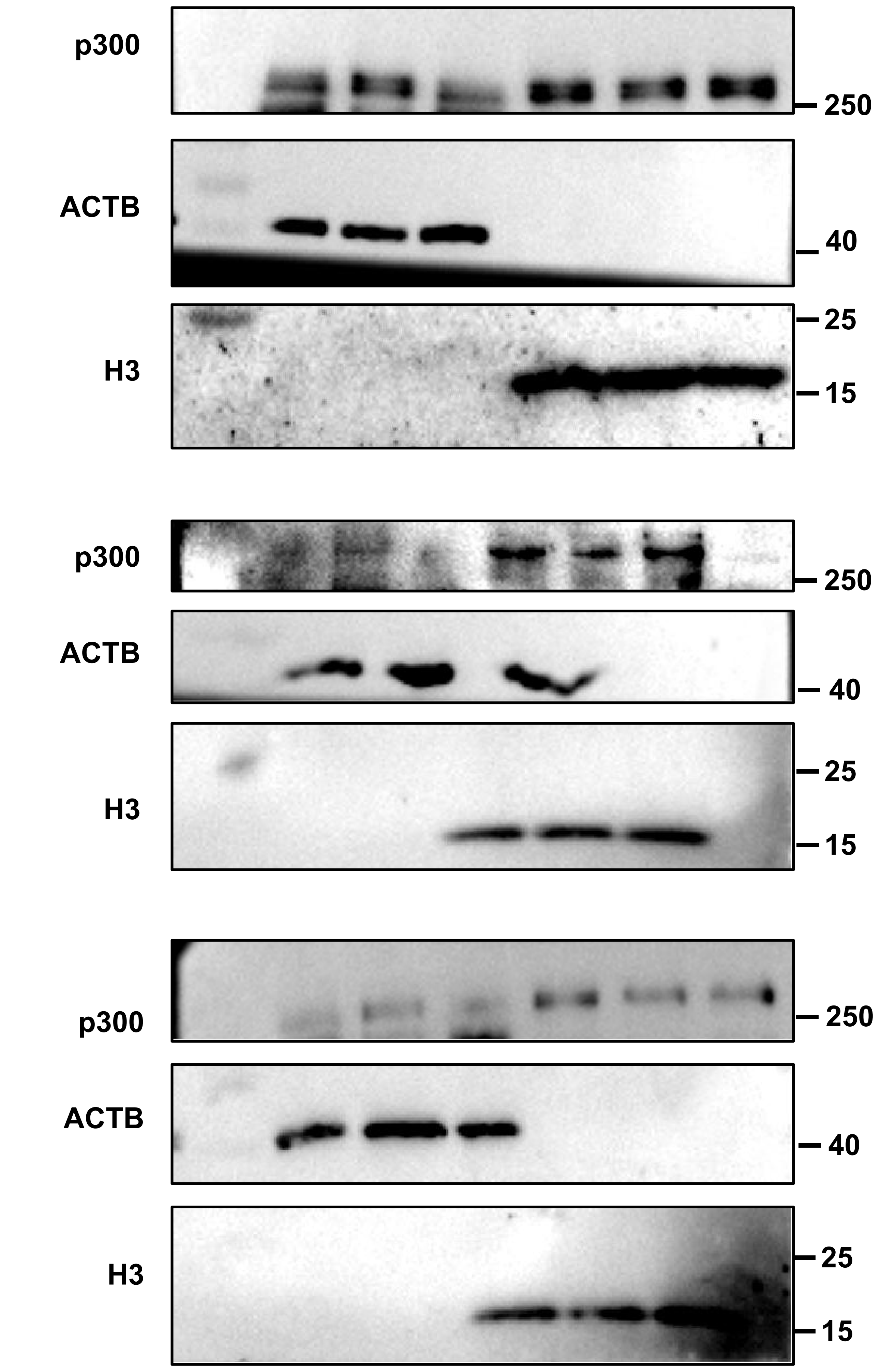

Source Data for Figure 6G

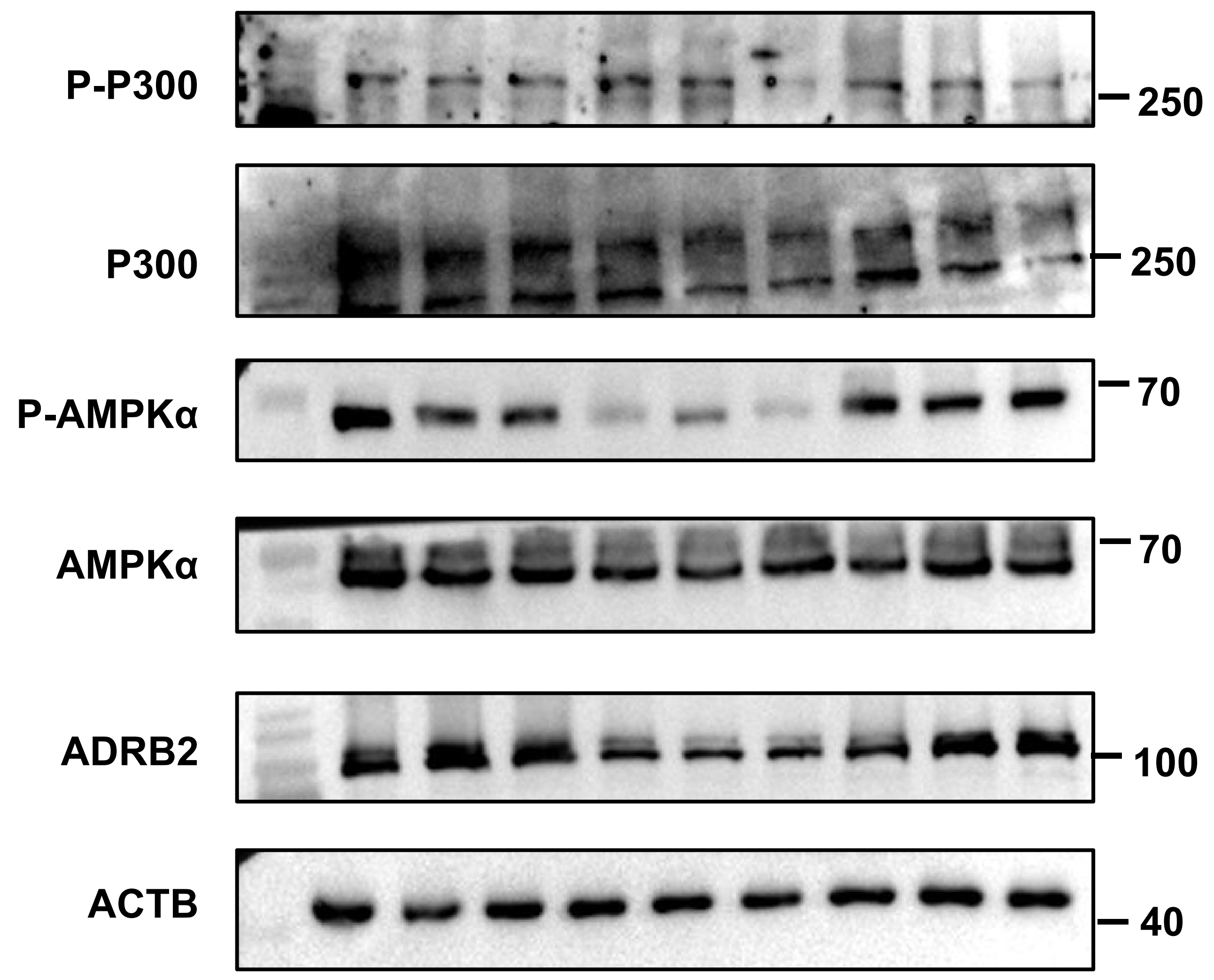

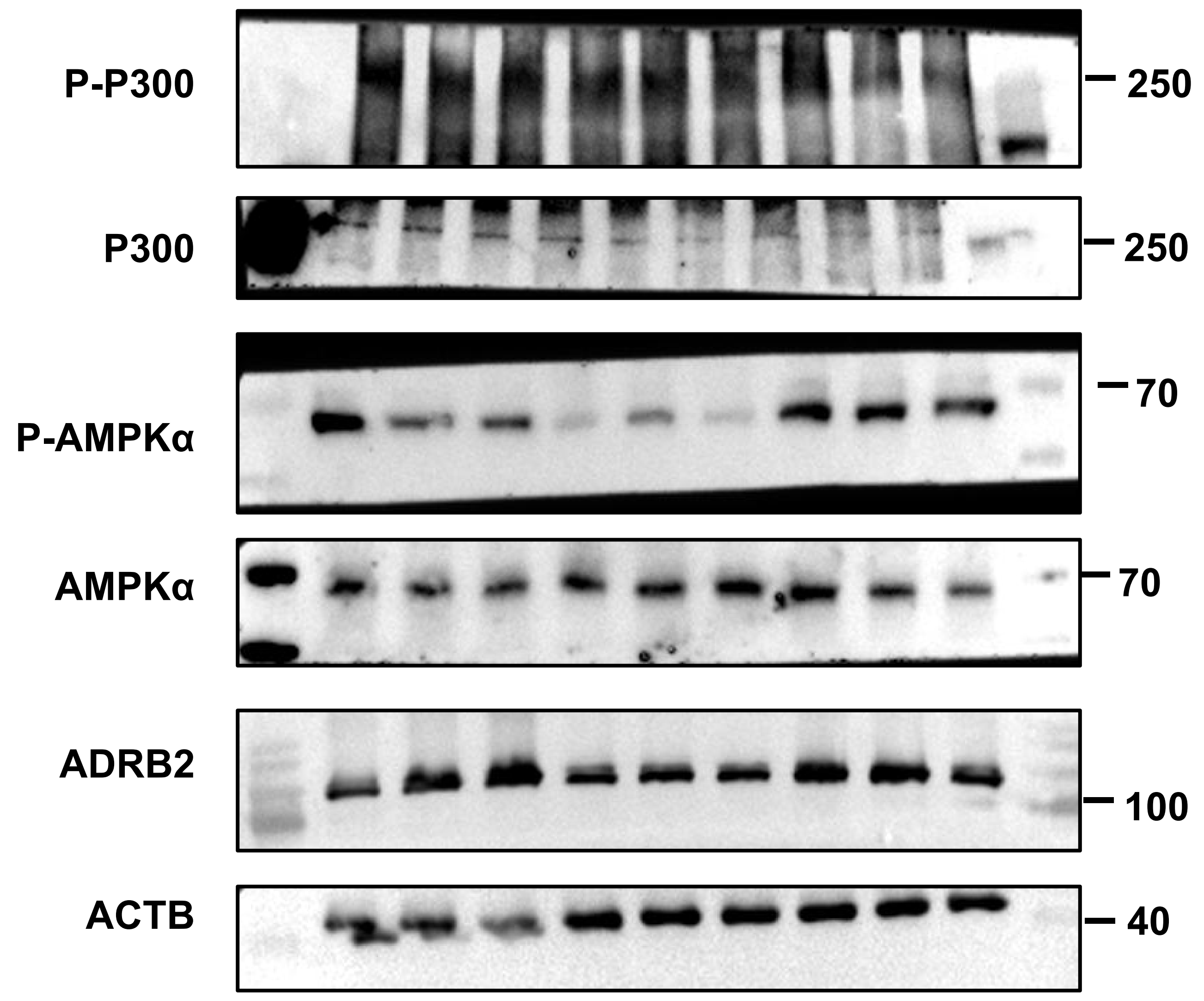

Source Data for Figure 6I

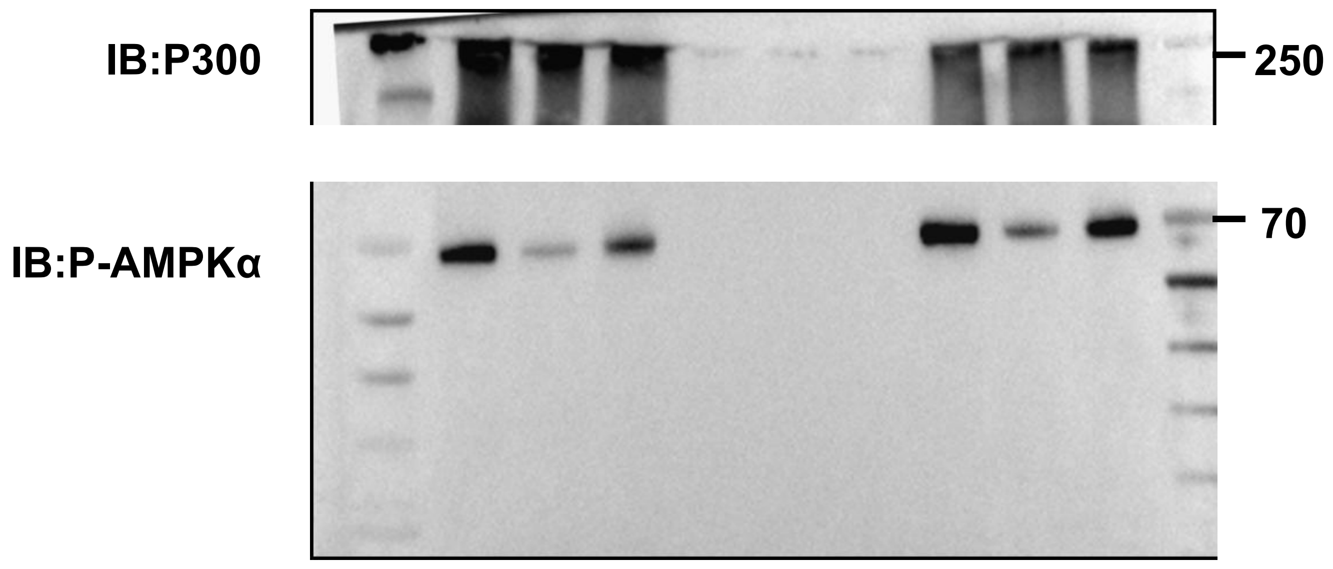

Source Data for Figure 6J

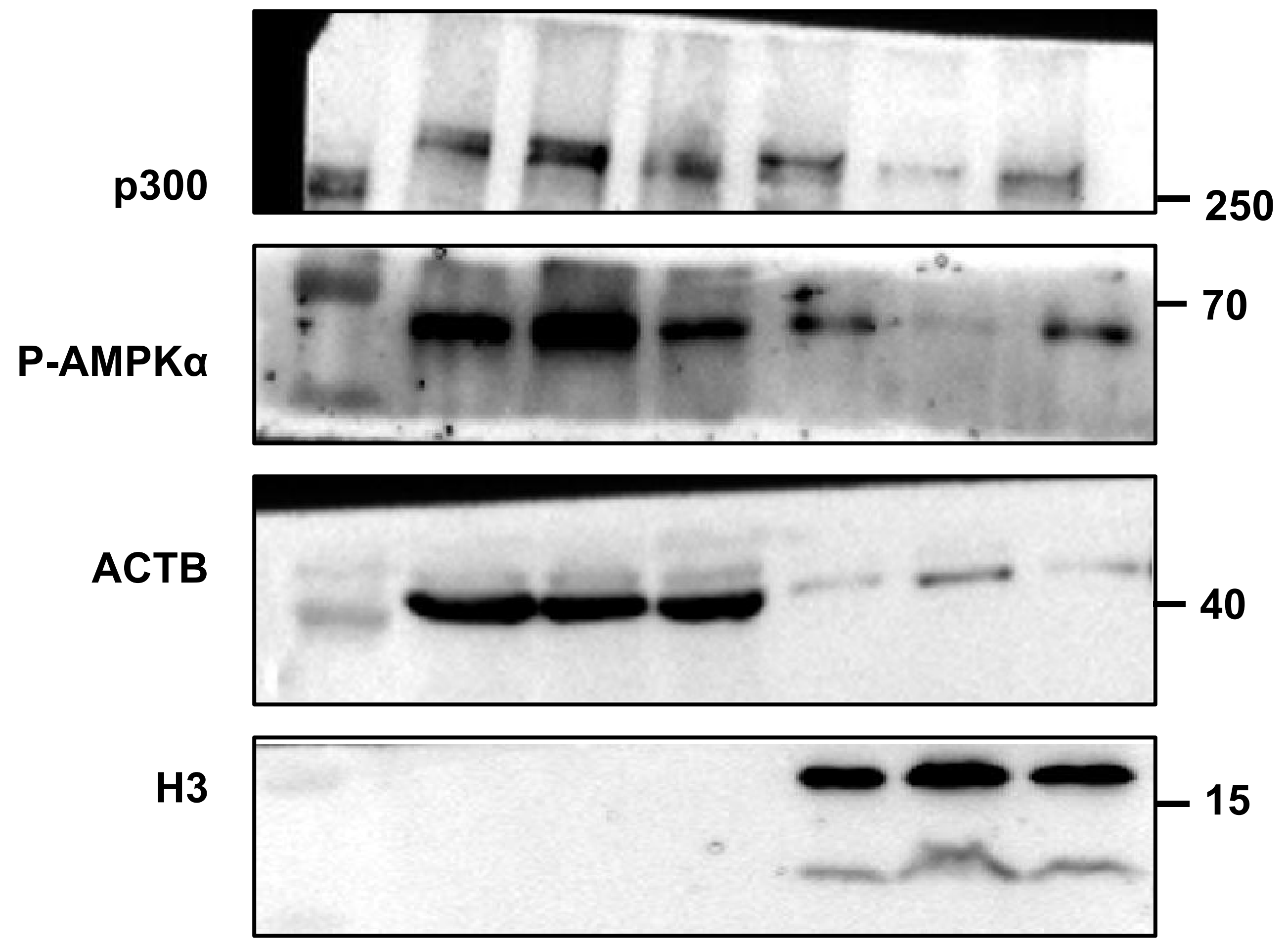

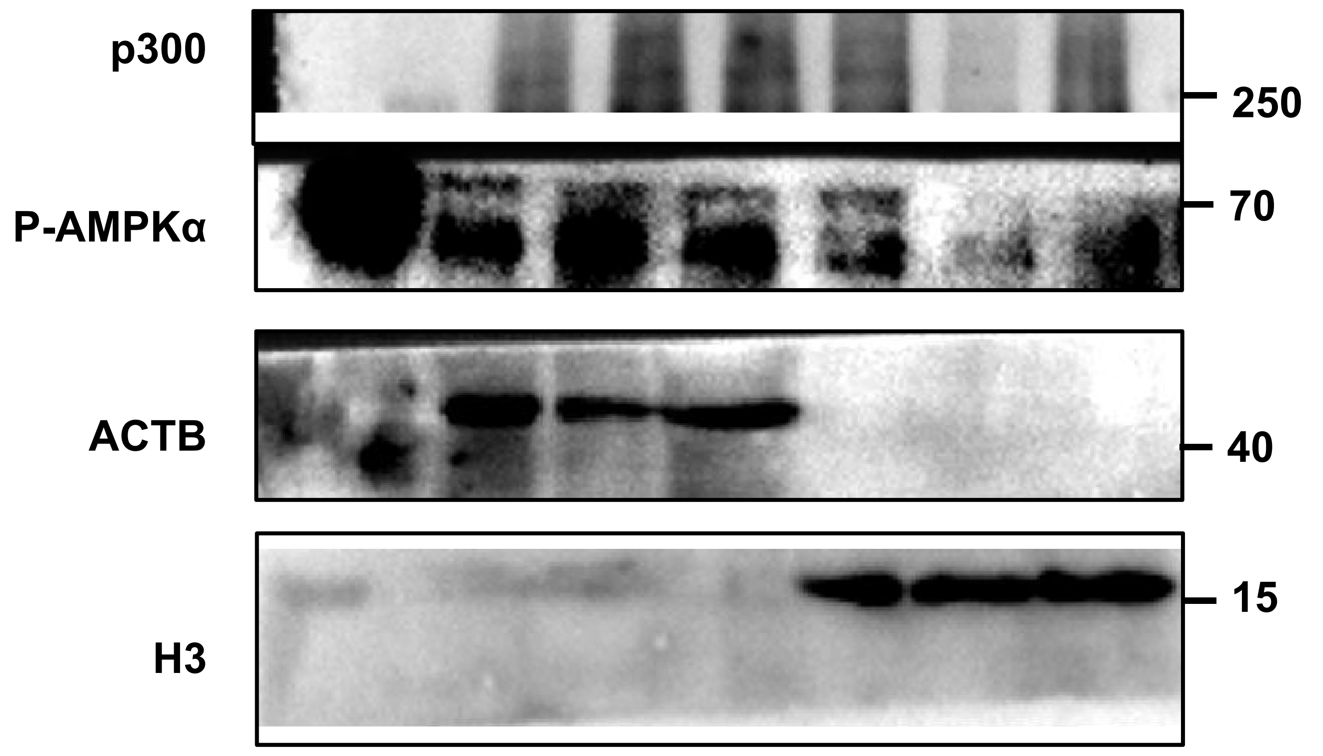

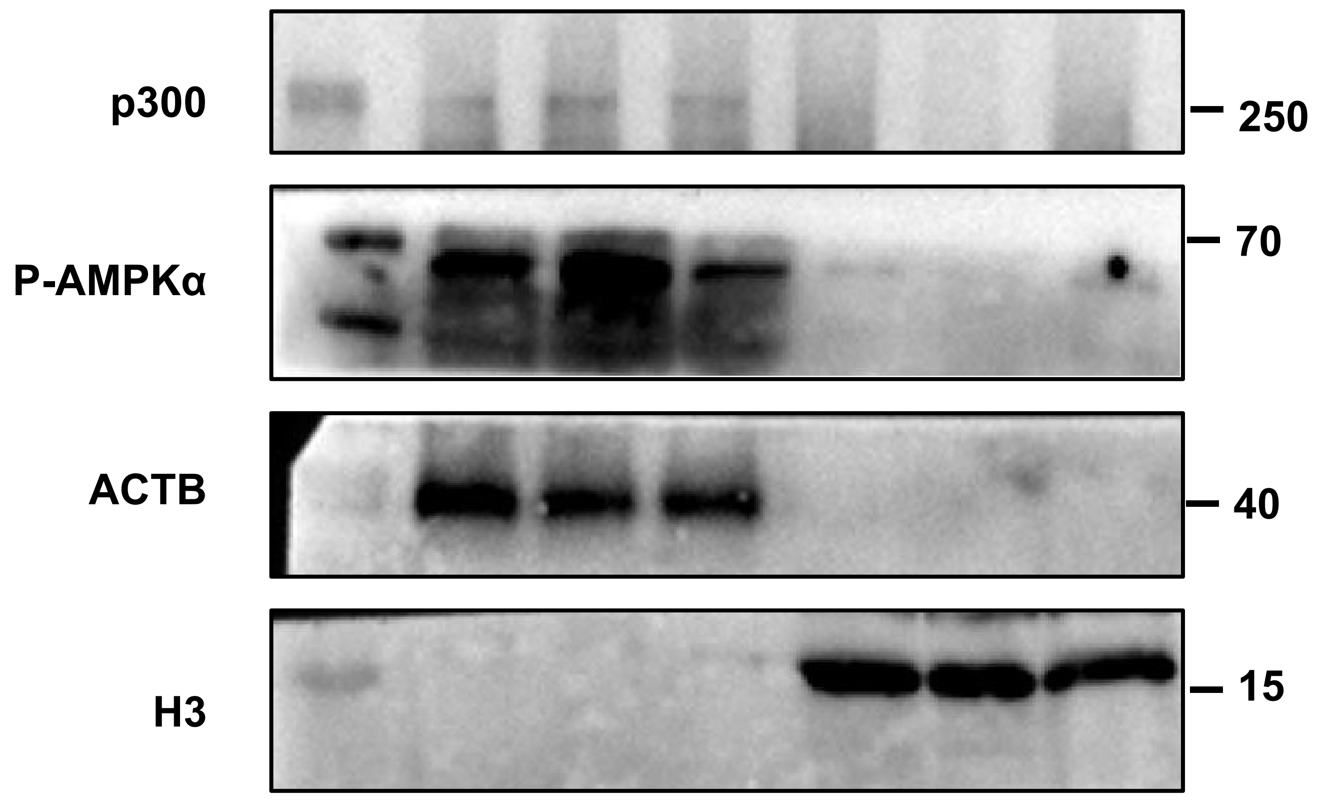

Source Data for Figure 6L

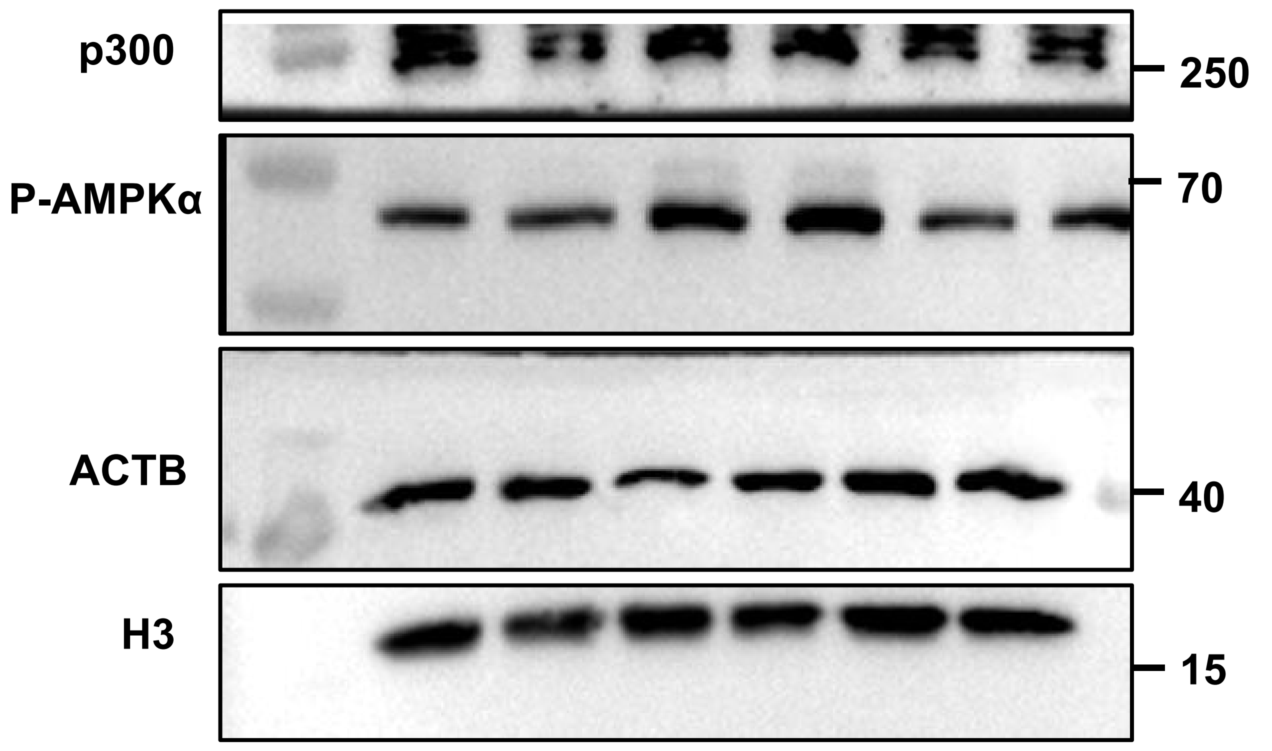

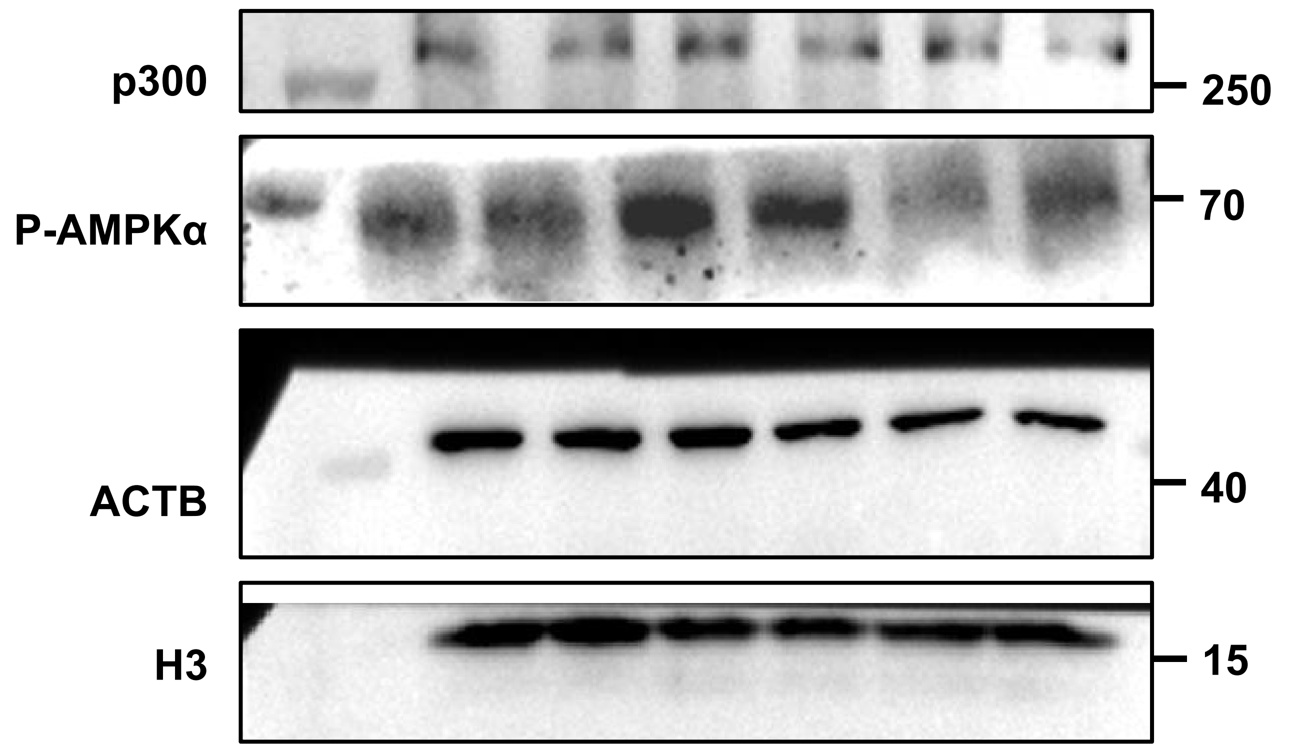

Source Data for Figure 6N

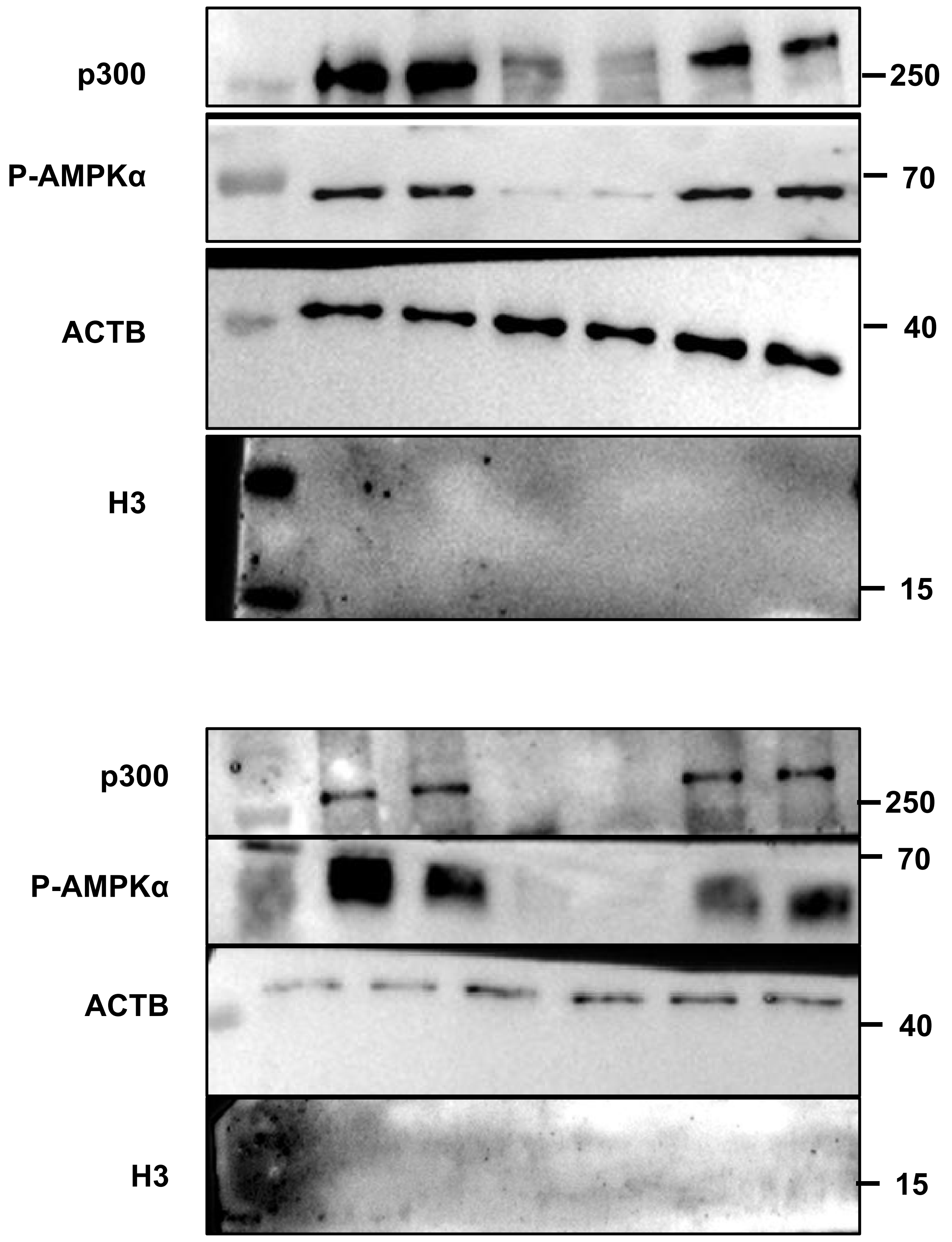

Source Data for Figure 6O

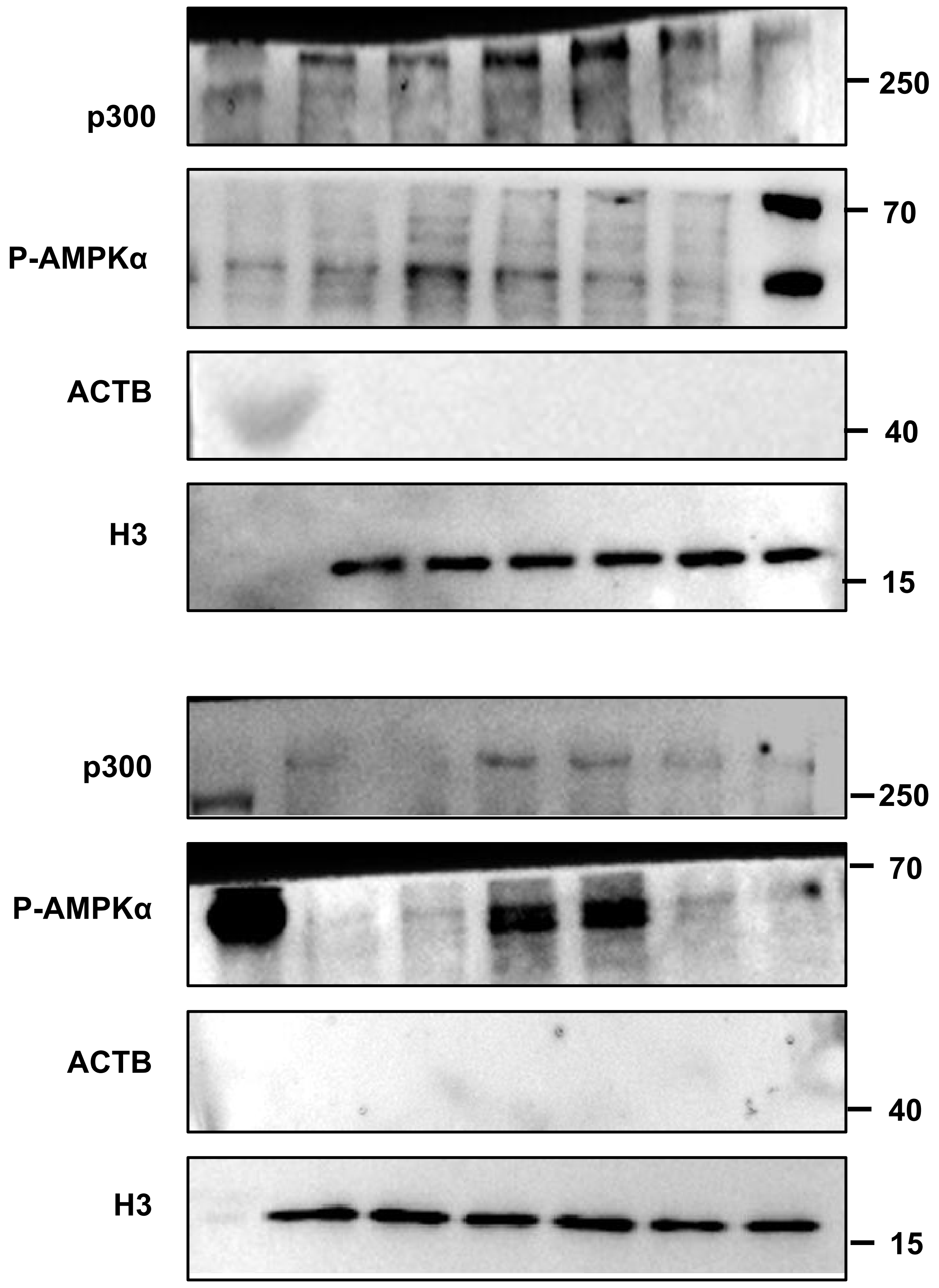

Source Data for Figure 6S

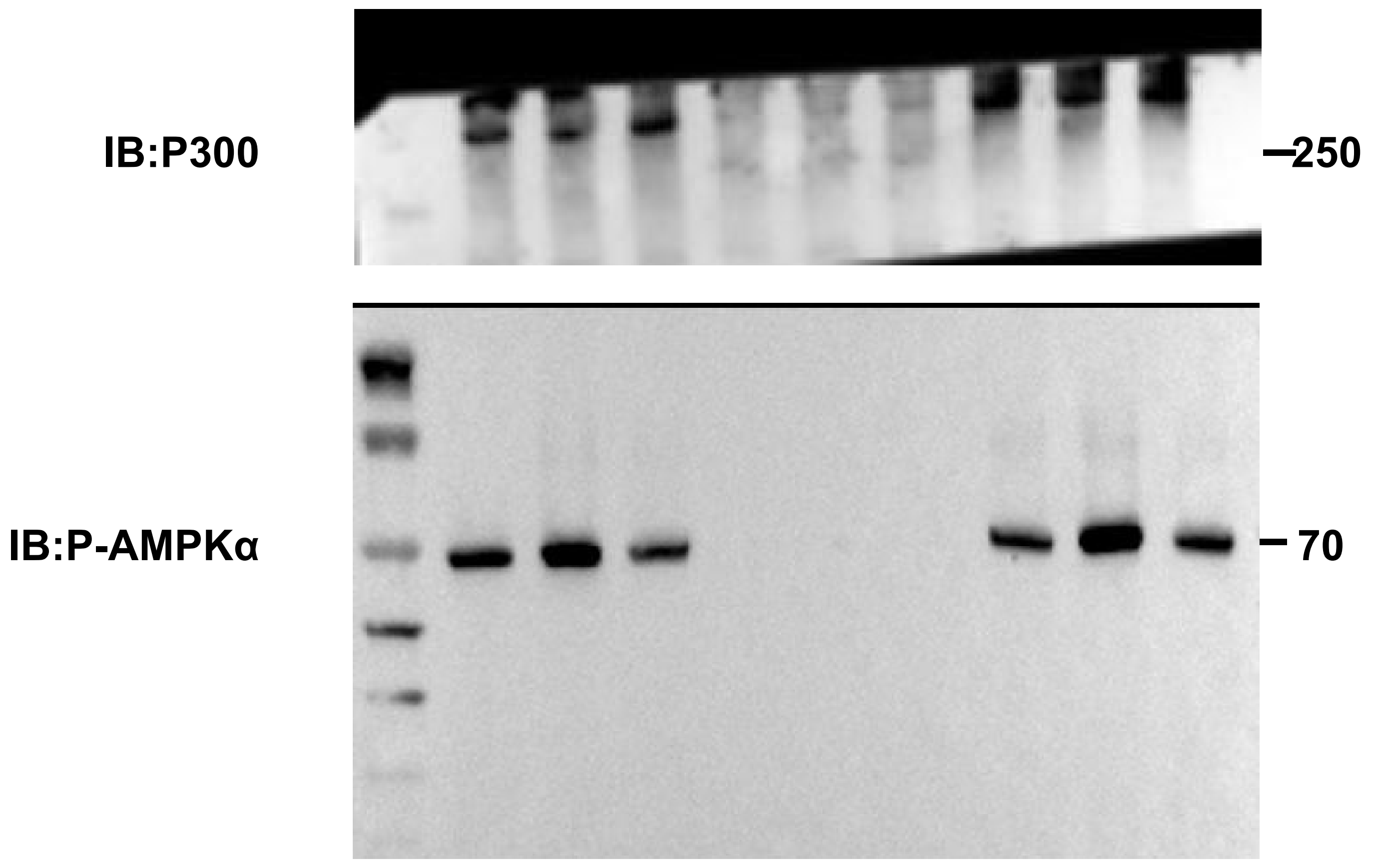

Source Data for Figure 7I

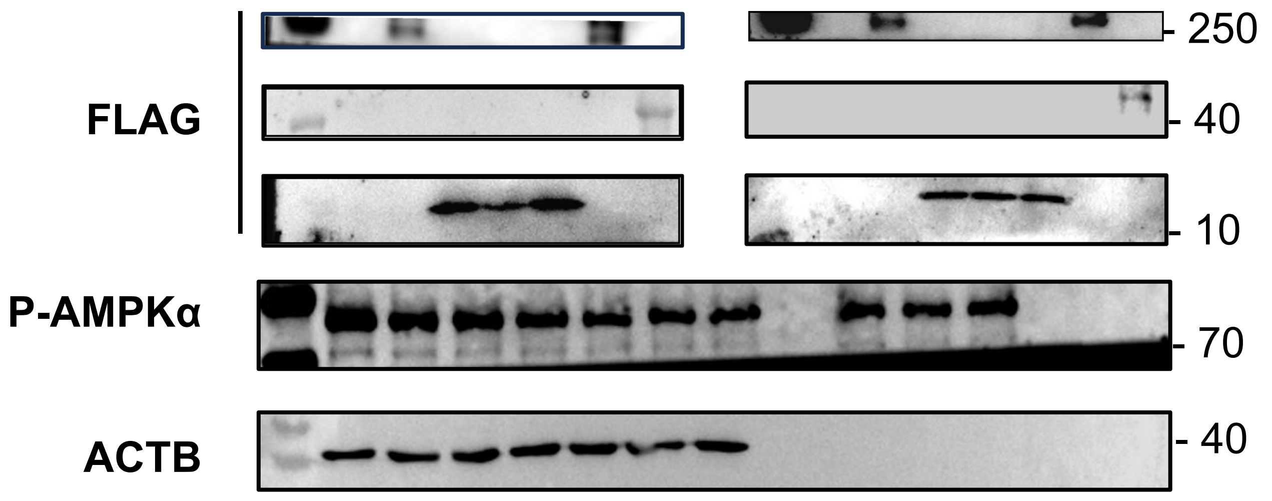

Source Data for Figure 7J

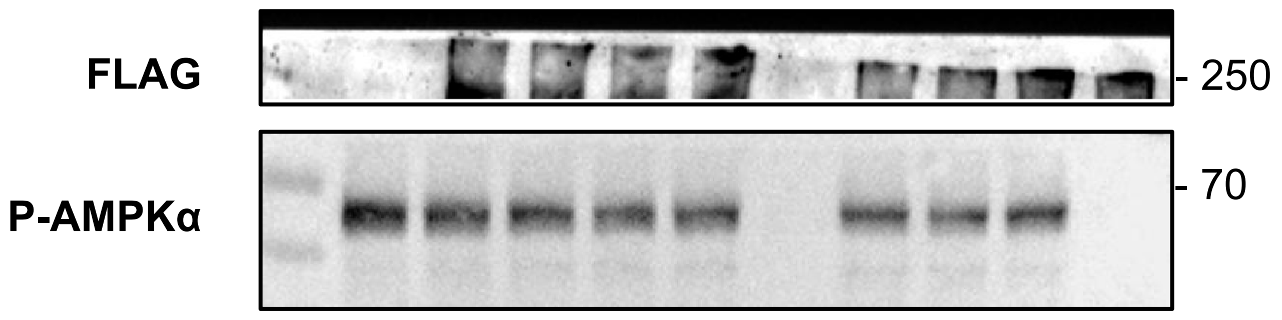

Source Data for Figure 7K

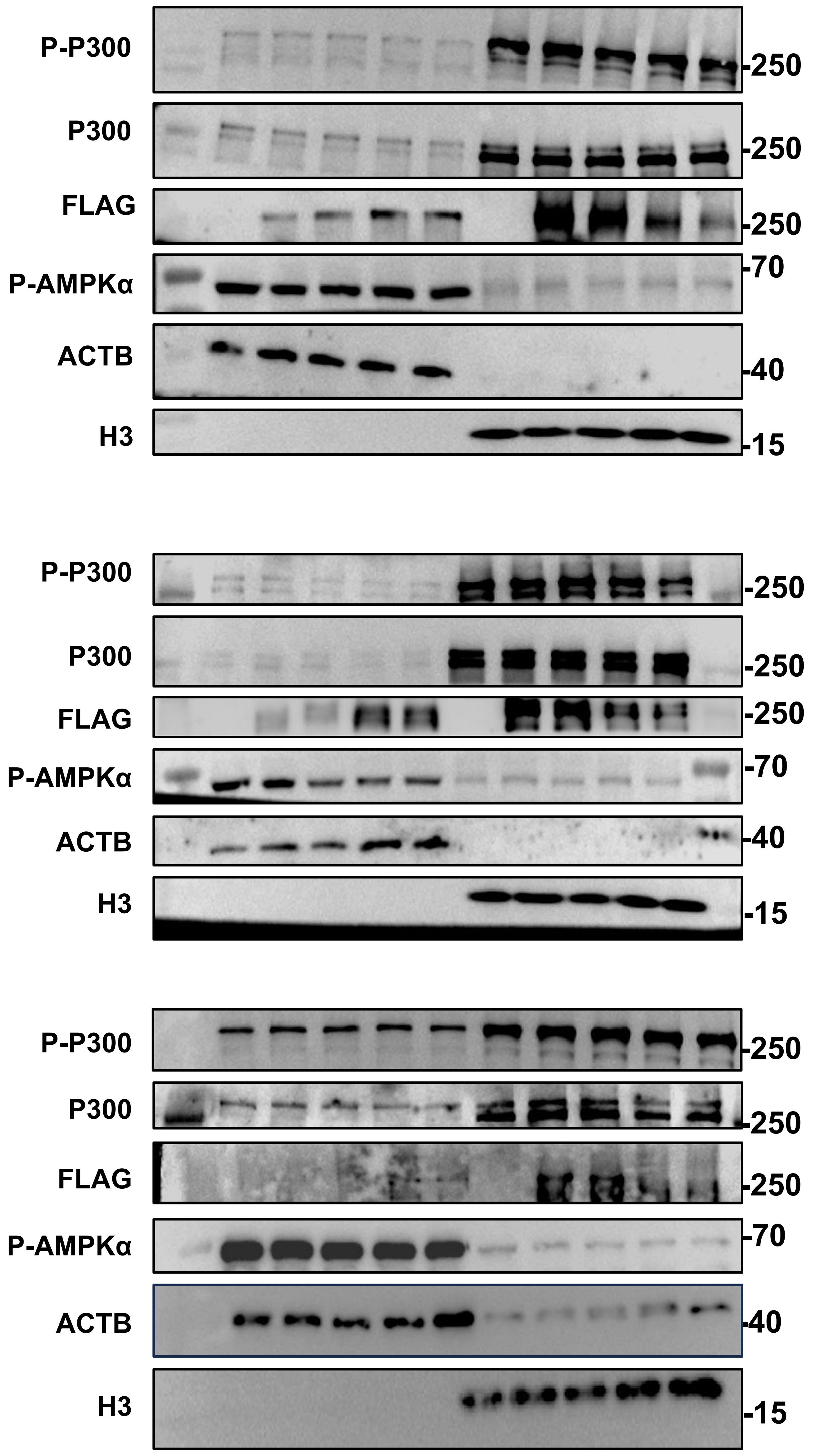

Source Data for Figure 7M

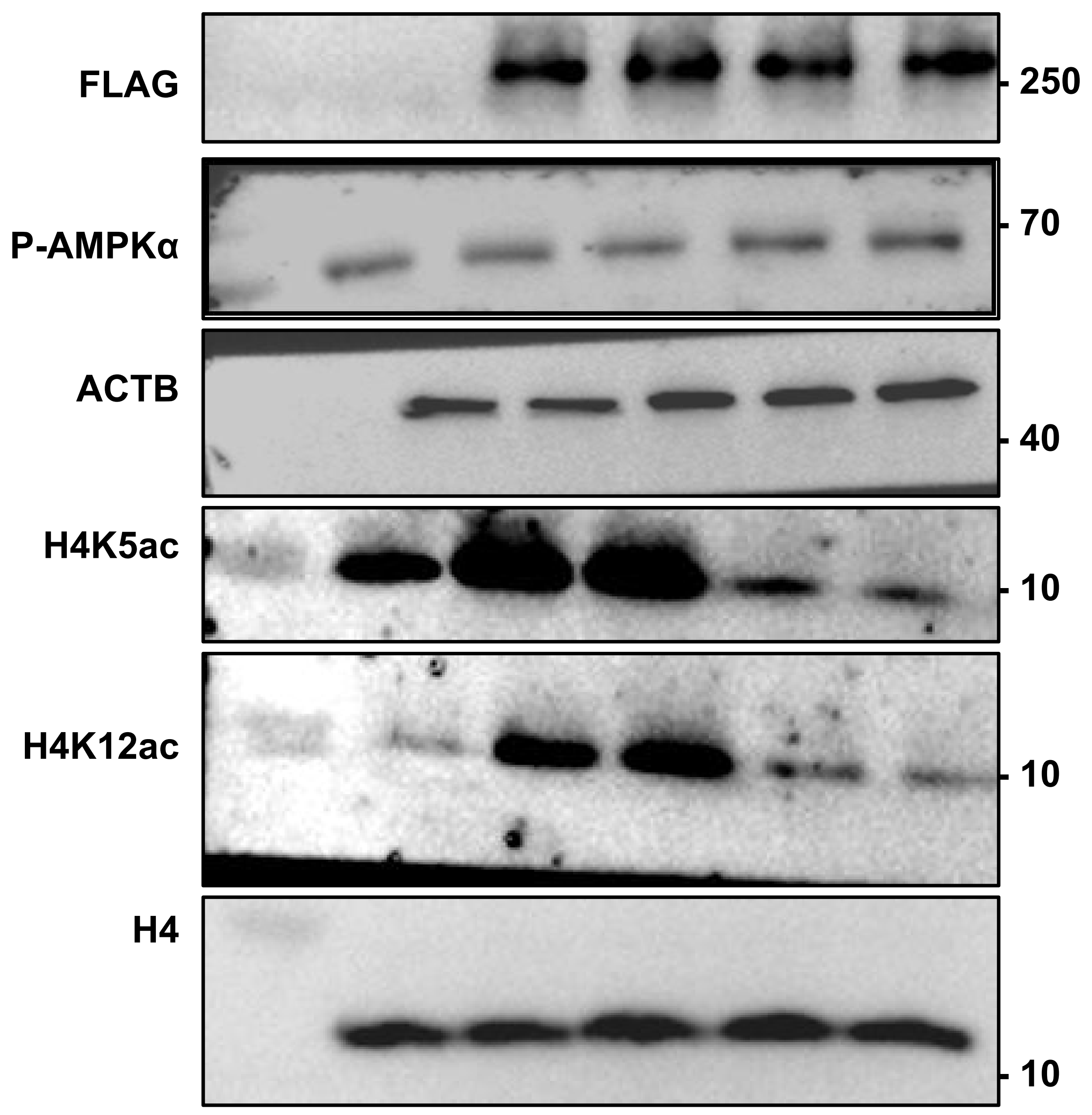

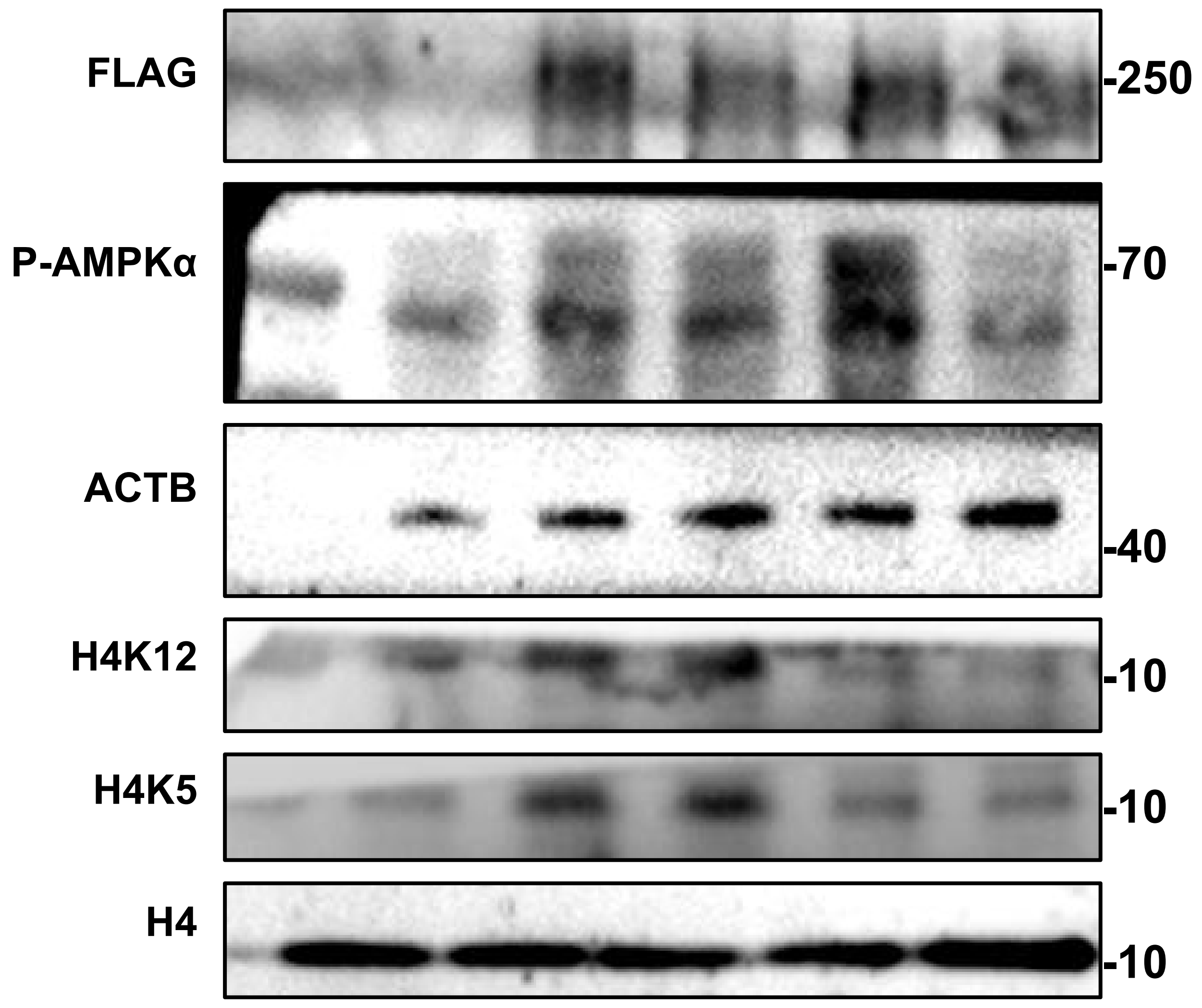

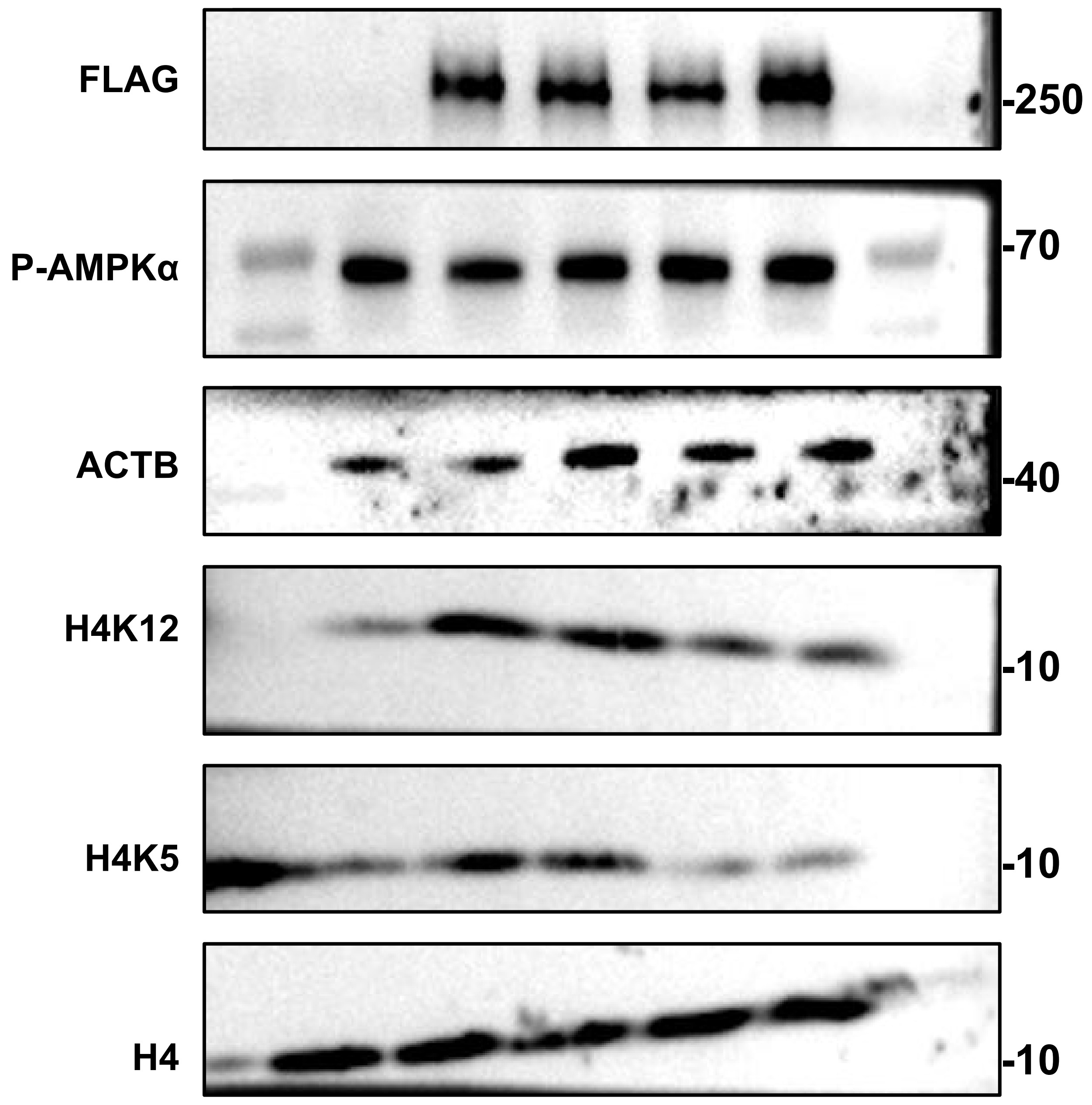

Source Data for Figure 8M

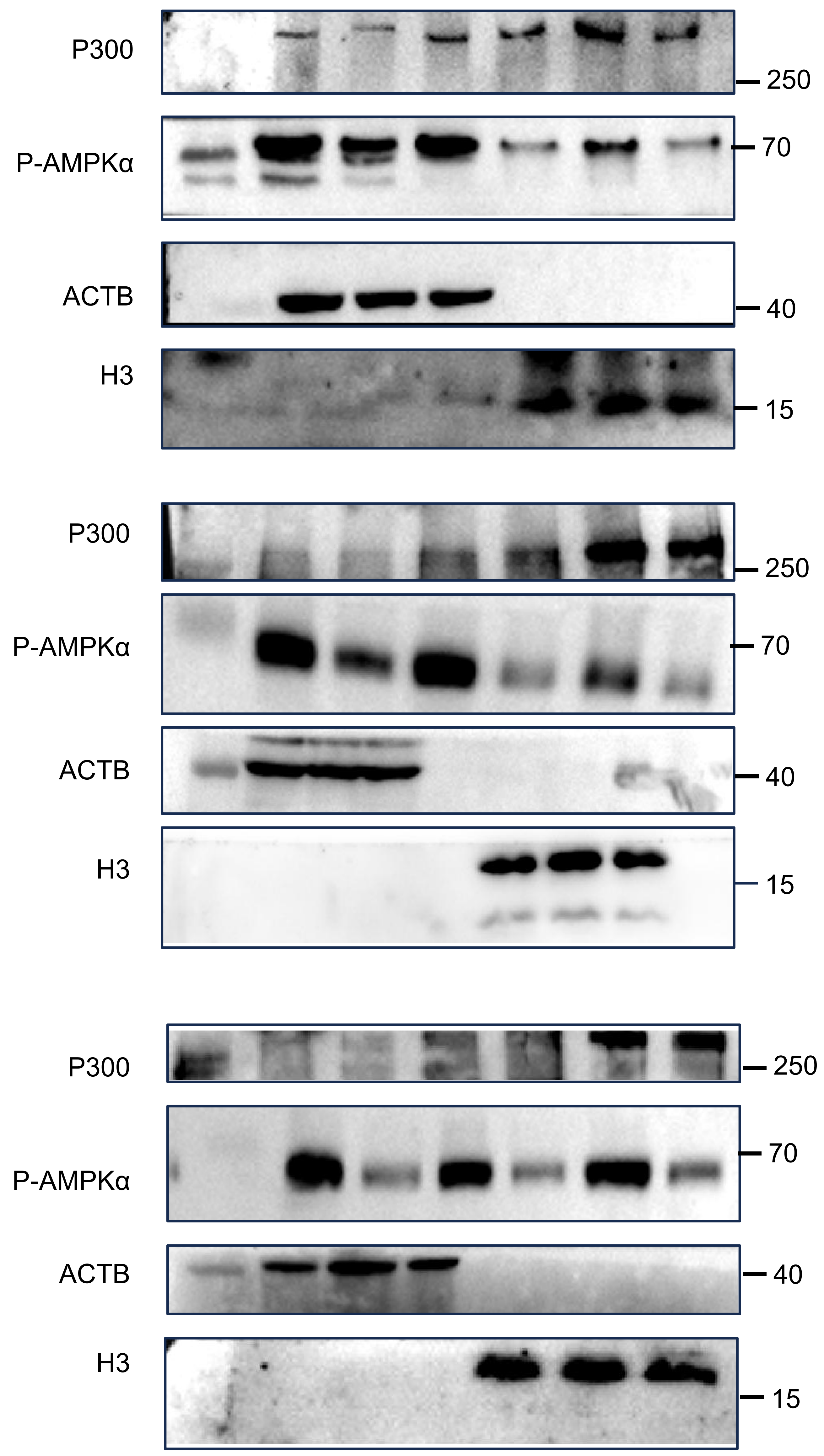

Source Data for Figure 8O

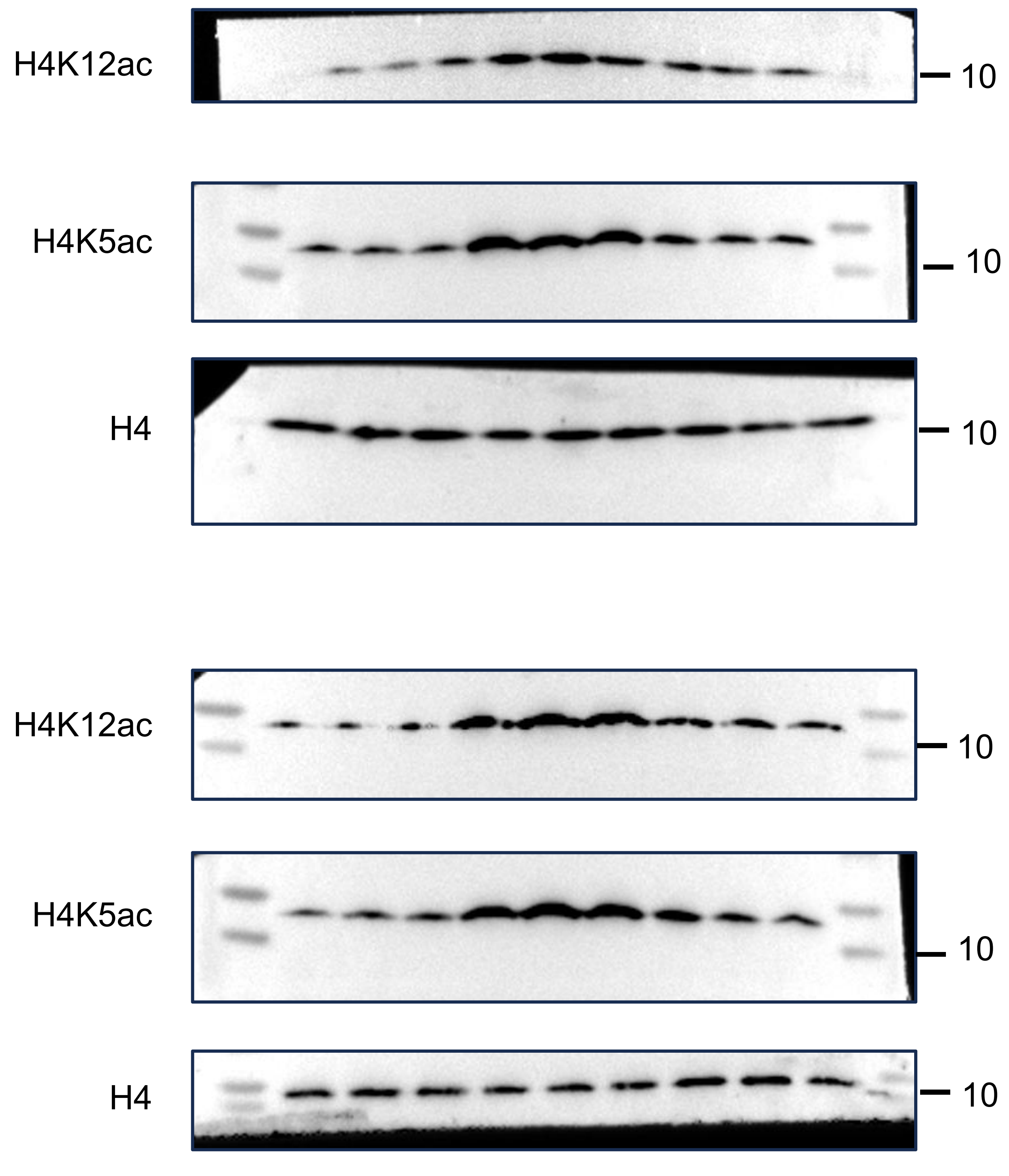

Source Data for Figure 8Q

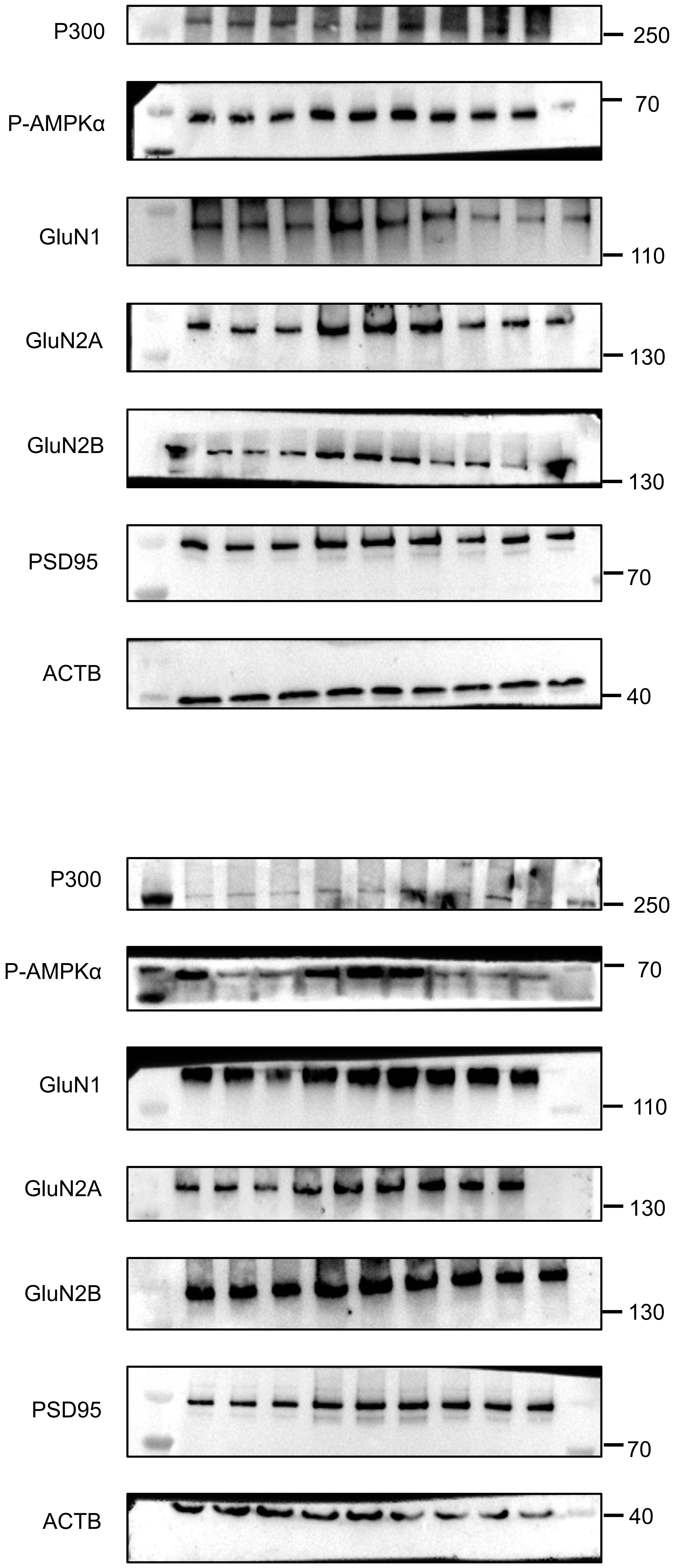

Supplement: Supplementary file 1 — Supplementary Material 1. [file 13195_2026_1983_MOESM1_ESM.pdf]
